# High-quality *Schistosoma haematobium* genome achieved by single-molecule and long-range sequencing

**DOI:** 10.1093/gigascience/giz108

**Published:** 2019-09-05

**Authors:** Andreas J Stroehlein, Pasi K Korhonen, Teik Min Chong, Yan Lue Lim, Kok Gan Chan, Bonnie Webster, David Rollinson, Paul J Brindley, Robin B Gasser, Neil D Young

**Affiliations:** 1 Department of Veterinary Biosciences, Melbourne Veterinary School, Faculty of Veterinary and Agricultural Sciences, The University of Melbourne, Corner Flemington Road and Park Drive, Parkville, VIC 3010, Australia; 2 Institute of Biological Sciences, Faculty of Science, University of Malaya, 50603 Kuala Lumpur, Wilayah Persekutuan Kuala Lumpur, Malaysia; 3 Parasites and Vectors Division, The Natural History Museum, Cromwell Rd, South Kensington, London SW7 5BD, UK; 4 School of Medicine & Health Sciences, Department of Microbiology, Immunology & Tropical Medicine, George Washington University, 2300 Eye Street, NW, Suite 502, Washington, DC 20037, USA

**Keywords:** *Schistosoma haematobium*, genome assembly, single-molecule and long-range sequencing

## Abstract

**Background:**

*Schistosoma haematobium* causes urogenital schistosomiasis, a neglected tropical disease affecting >100 million people worldwide. Chronic infection with this parasitic trematode can lead to urogenital conditions including female genital schistosomiasis and bladder cancer. At the molecular level, little is known about this blood fluke and the pathogenesis of the disease that it causes. To support molecular studies of this carcinogenic worm, we reported a draft genome for *S. haematobium* in 2012. Although a useful resource, its utility has been somewhat limited by its fragmentation.

**Findings:**

Here, we systematically enhanced the draft genome of *S. haematobium* using a single-molecule and long-range DNA-sequencing approach. We achieved a major improvement in the accuracy and contiguity of the genome assembly, making it superior or comparable to assemblies for other schistosome species. We transferred curated gene models to this assembly and, using enhanced gene annotation pipelines, inferred a gene set with as many or more complete gene models as those of other well-studied schistosomes. Using conserved, single-copy orthologs, we assessed the phylogenetic position of *S. haematobium* in relation to other parasitic flatworms for which draft genomes were available.

**Conclusions:**

We report a substantially enhanced genomic resource that represents a solid foundation for molecular research on *S. haematobium* and is poised to better underpin population and functional genomic investigations and to accelerate the search for new disease interventions.

## Background

Human schistosomiasis is a chronic, neglected tropical disease affecting >200 million people worldwide and resulting in >300,000 deaths each year [1]. *Schistosoma haematobium* (mainly in Africa), *Schistosoma mansoni* (mainly in Africa and South America), and *Schistosoma japonicum* (in Asia) are the 3 main blood flukes (schistosomes) of humans; the first causes urogenital schistosomiasis, and the other 2 cause hepatointestinal disease. Urogenital schistosomiasis results principally from a chronic (granulomatous) inflammatory process [2] directed at schistosome eggs entrapped in tissues [2–4] and is accompanied by increased risk for HIV/AIDS and infertility in women [5,6] and for squamous cell carcinoma of the urinary bladder [7]. Despite efforts to control schistosomiasis, it remains endemic in many subtropical and tropical regions of the world. Because there is no effective vaccine to protect humans [8], control currently relies heavily on targeted or mass treatment with the drug praziquantel [9], a reliance that risks the emergence of resistance to this compound [9]. In addition, treatment alone does not prevent reinfection. Thus, new, complementary interventions need to be established and implemented in the event that resistance to praziquantel becomes widespread [10], and to underpin efforts to eliminate the disease. Clearly, the development of interventions would be facilitated by sound knowledge and understanding of schistosome biology and the pathogenesis of the disease at the molecular level. However, fundamental and applied research on schistosomes has been neglected, particularly for *S. haematobium* [11], in spite of its high prevalence (>110 million people) in Africa. Since the London Declaration in 2012 [12], there has been an increased resolve by the scientific and philanthropic communities to tackle this problem [10].

In 2012, we reported a draft nuclear genome of *S. haematobium* (Egyptian strain, maintained at the Biomedical Research Institute, Rockville, Maryland [13]; NCBI:txid6185), assembled from short-read Illumina data derived from a single pair of adult worms [14]. This genome assembly enabled the inference of protein-encoding genes, functional annotation including gene ontology networks and metabolic pathways, and the exploration of the nature and extent of transposable elements [14]. Importantly, it also facilitated systematic comparative studies of genomes and gene families in human blood flukes [14–19]. In conjunction with other developments, including the establishment of a rodent model to study the pathogenesis for *S. haematobium* egg-induced disease [20], and knowledge that *S. haematobium* has a functional RNA interference pathway [21], the first draft genome for *S. haematobium* [14] has underpinned molecular investigations of schistosome biology, urogenital schistosomiasis [22], and associated cancer [23].

Despite the value of this resource for the schistosome research community, the utility of the draft genome assembly has been somewhat compromised by its fragmentation; the first assembly of the genome (designated Shae.V1) consisted of 99,953 contiguous sequences (i.e., scaffolds) that were interrupted by 29,422 gaps. Thus, the order and orientation of many segments of the genome could not be established. Genome finishing of large eukaryotic genomes using short-read sequence data is technically challenging, mostly due to difficulties assembling complex regions that are replete with dispersed repeats and large segmental duplications, which greatly complicates the determination of genome structure and sequence [24–26]. Subsequent annotation can be challenging due to complex and non-canonical gene structures [27]. In addition, gene prediction pipelines trained using data from model organisms are not accurate for divergent species [28]. Nevertheless, recently, advances in sequencing technologies have resulted in a systematic refinement of eukaryotic parasite genomes, enhanced gene sets, and an improved understanding of genomic architecture [29–31]. To complement these efforts and to provide an improved foundation for molecular research on *S. haematobium*, herein, we systematically improved the draft genome of *S. haematobium* by using a combination of single-molecule sequencing technology (Pacific Biosciences [PacBio]) [32], long-range (“Chicago”) library construction and Illumina sequencing, supported by existing Illumina short-read data [14]. On the basis of this enhanced reference, we refined the gene annotation, by transferring curated gene models from the original assembly and by using established gene (re-)annotation pipelines [25, 33] and published RNA-Seq data [14]. Subsequently, we re-assessed the phylogenetic position of *S. haematobium* relative to other trematodes, for which draft genomes were publicly available, using amino acid sequence data sets inferred from single-copy orthologs shared among all taxa included in the analysis.

## Data Description

### Sample procurement, preparation, and storage

All samples originated from the same Egyptian strain of *S. haematobium* that was used to assemble the first draft genome of *S. haematobium* [14]. This strain is maintained at the Biomedical Research Institute, Rockville, Maryland [13], in *Bulinus truncatus* (intermediate snail host) and *Mesocricetus auratus* (hamster; mammalian definitive host). Hamsters were each infected with 1,000 cercariae. Ninety days later, paired adults of *S. haematobium* were collected from *M. auratus*, following the perfusion of the mesenteric and intestinal vessels using physiological saline (37°C). Worms were prepared and stored as previously described [14].

### Single-molecule and long-range library construction and genomic sequencing

For long-read sequencing (PacBio), genomic DNA (∼1 μg) was isolated from a single pair of adult worms (i.e., male and female *in copula*; isolate MP2018; BioSample ID: SAMN10797288) of *S. haematobium* using a kit (Chemagic DNA Tissue Extraction Kit, Chemagen, Baesweiler, Germany), and 25 ng were subjected to whole-genome amplification using a REPLI-g Single Cell Kit (Qiagen, Hilden, Germany). The amplified DNA was purified and concentrated using 0.45-fold volume of Agencourt AMPure XP magnetic beads (Beckman Coulter, Brea, CA, USA). DNA amount was determined using a Qubit fluorometer dsDNA HS Kit (Life Technologies, Carlsbad, CA, USA), and its integrity was verified by agarose gel electrophoresis. Whole-genome amplification DNA (8 µg) was sheared to ∼10 kb using a g-TUBE (Covaris, Woburn, MA, USA), purified and concentrated using 0.45-fold volume of washed Agencourt AMPure XP magnetic beads (Beckman Coulter), and examined using an Agilent 2100 Bioanalyzer (Agilent Technologies, Santa Clara, CA, USA). This sheared DNA was used to construct a SMRTbell library (∼2.7 kb average size) using the SMRTbell Template Preparation Kit (v.1.0; PacBio, Menlo Park, CA, USA). In brief, sheared DNA was subjected to end repair, ligation of adaptors, and exonuclease digestion of incomplete SMRTbell templates. Thereafter, library sequencing primers were annealed (0.83 nM final concentration) to the SMRTbell template, allowing the P4 DNA polymerase (DNA Polymerase Binding Reagent Kit; PacBio) to bind. This complex was immobilized on Magbeads (PacBio) using protocols for enhanced loading efficiency. Sequencing was performed on the PacBio RS II system (PacBio) using 33 single-molecule real-time (SMRT) cells and Sequencing Reagent 2.0 (PacBio). Sequence data were collected using a 180-minute movie length and the stage-start option. Adaptors, short reads (<50 bases), and nucleotides with an estimated polymerase read quality value of <0.75 were removed from the acquired data using the SMRT analysis software (v.2.1.0.0.127824; PacBio).

To construct a long-range (“Chicago”) linking library, used for scaffolding [34], genomic DNA was isolated from ∼20 pairs of adult worms (i.e., male and female *in copula*; BioSample ID: SAMN10797287) of *S. haematobium* using a Chemagic DNA Tissue Extraction Kit. In brief, genomic DNA (2 μg) was fragmented to produce 500 ng of high-molecular-weight DNA (mean fragment size: 50 kb), which was reconstituted into chromatin *in vitro* and fixed with formaldehyde. Fixed chromatin was digested with DpnII, the 5´-overhangs were filled in with biotinylated nucleotides, and the free blunt ends were ligated. After ligation, cross-links were reversed, and the DNA was purified from protein. Purified DNA was treated to remove biotin that was not internal to ligated fragments. The DNA was sheared to a mean fragment size of ∼350 bp, and sequencing libraries were constructed using NEBNext Ultra enzymes and Illumina-compatible adaptors. Biotin-containing fragments were isolated using streptavidin beads before PCR-based enrichment of the library. This library was sequenced (100 bp, paired-end reads) using an Illumina HiSeq 2500 platform following the rapid-run protocol.

### Pre-assembly processing of sequence data

First, SMRTbell adaptors were removed from PacBio reads using BBMAP (v.37.33, RRID:SCR_016965) [35]. Due to a 19-fold coverage, these reads were error-corrected using the program LoRDEC (v.0.3, RRID:SCR_015814; "correct" and "trim" options) [36]. Second, using existing Illumina paired-end, short-insert libraries (170, 500, and 800 bp; NCBI BioProject accession number: PRJNA78265) [14], a de Bruijn graph with *k*-mers of length 21 was generated. Third, low-quality bases (Phred quality score <25), adaptors, and reads of <40 nucleotides in length were removed from long-range sequence data, using the program Trimmomatic (v.0.32, RRID:SCR_011848) [37].

### Genome assembly

The new genome scaffolds (designated Shae.V2 genome scaffolds) were assembled in a stepwise manner:

1. All contigs making up the Shae.V1 assembly (*n* = 129,375) [14] were scaffolded using long-range, paired-read data using the Dovetail HiRise pipeline (v.2.0.5) [34]. In brief, reads were aligned to contigs using SNAP-align (v.1.0dev.67_as) [38], masking out bases that follow a junction of 2 sites of recognition for the restriction enzyme MboI (GATCGATC), and removing the penalty assigned to the map quality for any 2 reads that formed a pair but mapped to different scaffolds. To identify repetitive genomic regions, 500-bp reads from a previous study [14] were aligned to the Shae.V1 contigs using SNAP-align. All alignment files were compressed into the BAM format, sorted, and indexed using the program SAMtools (v.1.6–7-g35457e2, RRID:SCR_002105) [39]. Duplicates were removed using the sorted BAM files and Picard tools (v.1.123) [40]. Subsequently, the HiRise pipeline was used to iteratively identify and break mis-assemblies and rescaffold contigs using an established method [34].
2. The Haplomerger2 pipeline (v.3.2) [41] was used to remove redundancy in scaffolds of >250 bp in length that were generated by HiRise, to improve scaffolding using published 2, 5, and 10 kb Illumina mate-pair libraries [14] and to close gaps in scaffolds using published 170, 500, and 800 bp paired-end, short-read libraries [14].
3. Corrected PacBio reads were used to close gaps in scaffolds using PBJELLY2 (PBSuite v.14.9.9) [42].
4. Corrected PacBio long-read data were also used to improve the assembly of scaffolds using SSPACE-LongRead (v.1.1) [43], requiring 3 links between scaffolds.

Following assembly, “contaminant” scaffolds with homology to bacteria but without nucleotide sequence homology to schistosome scaffolds were identified by searching the NCBI nt database [44] using BLASTn (v.2.5.1+, RRID:SCR_001598) [45] and removed. The completeness of the Shae.V2 genome assembly was assessed using BUSCO (v.3.0, RRID:SCR_015008) [46] in the genome mode, and compared with BUSCO results for the published Shae.V1, *S. japonicum*, and *S. mansoni* assemblies [14, 29, 47]. The lengths and locations of ambiguous nucleotide homopolymer gaps were assessed in each set of genome scaffolds using SeqKit (v.0.6.0) [48]. The coverage of individual Shae.V2 genome scaffolds was assessed by mapping short-insert (insert size: 170 and 500 bp), mate-pair (800 bp and 2, 5, and 10 kb), Chicago long-range, and PacBio reads to the assembled scaffolds using SNAP-align (for Illumina reads) or BLASR (for PacBio reads; v.2.2.0.133377, RRID:SCR_000764) [49]. Alignment results were filtered for “properly mapped pairs” (using "samtools view," -f2 option) and then stored and sorted in the BAM format. Sorted BAM files were merged, and coverage was determined using "samtools depth" (read coverage) and "bamCoverage" (v.3.0.1; -e option; “physical” coverage, considering regions spanned by paired-end reads as covered) [50], respectively. Regions of >1,000 nucleotides were designated as “regions of low coverage” if <5 reads (for read coverage) or <10 reads (for “physical” coverage) mapped.

### Transfer of existing gene models to newly assembled scaffolds and prediction of a final gene set

Existing protein-encoding gene models for the Shae.V1 gene set [14], stored in the general feature format (GFF), were transferred to the Shae.V2 scaffolds using liftOver (kentUtils v.302) [51] and RATT (v.0.95) [52]. The Shae.V1 gene set included manually or semi-automatically curated gene models published in earlier studies, including those for G-coupled protein receptors (GPCRs) [18], protein kinases [19], annexins [16], and SCP/TAPS [15]. For liftOver, an available repeat library [14] was used to soft-mask both Shae.V1 and Shae.V2 scaffolds using RepeatMasker (v.4.0.5, RRID:SCR_012954) [53]. LASTZ (v.1.02.00) [54] and chainNet tools (jksrc20100603 within Haplomerger2 v.3.2) [55] were used to identify aligned “blocks” in each set of scaffolds. Within aligned blocks, genes from the Shae.V1 gene set were transferred to respective Shae.V2 scaffolds using liftOver. In addition, soft-masked Shae.V1 and Shae.V2 scaffolds and the Shae.V1 gene set were used to transfer protein-encoding gene models to Shae.V2 using RATT. Transferred gene models were stored in the GFF format for further processing.

A final Shae.V2 gene set was inferred by combining gene models transferred from Shae.V1, gene model-evidence derived from transcriptomic data (RNA-Seq), *ab initio* gene predictions, and evidence of genomic regions encoding proteins homologous to predicted proteins in other flatworms using the programs MAKER2 (v.2.3.8) [56] and EVM (v.1.1.1) [57] in a stepwise manner:

1. Available RNA-Seq data for adult (male and female) and egg stages [14] was assembled *de novo* using Trinity (v.2.2.0, RRID:SCR_013048) [58]. Assembled, non-redundant, full-length transcripts were predicted using TransDecoder (v.2.1.0) [59].
2. Available RNA-Seq data were mapped to Shae.V2 genome scaffolds using TopHat2 (v.2.1.0) [60], and gene models were inferred from mapped RNA-Seq data using Cufflinks (v.2.2.1, RRID:SCR_014597) [61].
3. Gene models were predicted *ab initio* using AUGUSTUS (v.3.1, RRID:SCR_008417) [62], SNAP (v.6.7) [63], and GENEMARK (v.4.2.9, RRID:SCR_011930) [64] with full-length, *de novo*–assembled transcripts (step 1) used for gene model training.
4. *Ab initio*gene predictions, gene models inferred from RNA-Seq data, non-redundant transcriptomes, transferred Shae.V1 gene models, and genome-aligned predicted proteomes for *S. mansoni* (NCBI BioProject: PRJEA36577) [29] and *S. japonicum* (NCBI BioProject: PRJEA34885) [47] were combined in MAKER2 to create a gene set.
5. EVM was used to select reliable gene models by using modelled gene structures inferred using the *de novo*–assembled transcriptome and PASA2 (v.2.0.2) [65], and by incorporating all gene model evidence inferred by MAKER2.

Concatenated GFF files from MAKER2, EVM, RATT, and liftOver were compared to identify overlapping gene models using GFFREAD (v.2.2.1) [66] using the merge (–m) option. If gene models overlapped with existing Shae.V1 genes, the model with the longest open reading frame (ORF) was defined as being representative and thus retained. Gene models for Shae.V1 that could not be transferred to the Shae.V2 genome using liftOver or RATT were identified by matching them with the most similar gene in the gene sets inferred using MAKER2 and EVM, of which the longest ORF representing the coding region was retained.

Proteins inferred from the merged gene model files that were similar to the Shae.V1 gene set were compared with the predicted proteome of *S. mansoni* using OrthoMCL (v.2.0.4) [67]. *Schistosoma mansoni* proteins that had no predicted ortholog in the transferred Shae.V1 gene set but shared amino acid sequence similarity (BLASTp; v.2.5.1+) [45] with predicted proteins in gene models inferred using MAKER2 and/or EVM were identified. For these gene models, the longest ORF encoding the respective protein sequence was retained in the final gene set.

All retained gene models were merged into a single GFF file. Subsequently, their integrity was confirmed and overlapping gene models were removed using GAG (v.2.0.1) [68] and tbl2asn (v.25.3, RRID:SCR_016636) [69]. The completeness of the final gene set was assessed by searching for orthologs of 978 conserved gene models representing metazoans, using the program BUSCO in the gene set mode. For comparisons among gene sets, the same analysis was carried out for Shae.V1, *S. mansoni*, and *S. japonicum* gene sets (WormBase Parasite version WBPS8).

### Determining synteny between genomes

Proteins predicted from the Shae.V1 and Shae.V2 and *S. mansoni* genomes were compared using OrthoMCL, and inferred single-copy orthologs (SCOs) were selected for further processing. The number and order of syntenic blocks containing ≥3 SCOs was assessed using OrthoCluster [70]. Syntenic scaffolds and comparisons of assembly contiguity and integrity between genomes were displayed as circular plots using Circos (v.0.69–6, RRID:SCR_011798) [71] and edited using Inkscape (RRID:SCR_014479) [72].

### Phylogenetic analysis

Single-copy orthologous groups of genes (*n* = 410) shared among 14 trematode species (*S. haematobium*, PRJNA78265; *Schistosoma bovis*, PRJNA451066 [73]; *Schistosoma curassoni*, PRJEB519; *Schistosoma mattheei*, PRJEB523; *Schistosoma margrebowiei*, PRJEB522; *S. mansoni*, PRJEA36577; *Schistosoma rodhaini*, PRJEB526; *S. japonicum*, PRJEA34885; *Trichobilharzia regenti*, PRJEB4662; *Clonorchis sinensis*, PRJNA386618; *Opisthorchis viverrini*, PRJNA222628; *Paragonimus westermani*, PRJNA454344 [74]; *Fasciola hepatica*, PRJNA179522; and *Echinostoma caproni*, PRJEB1207), for which draft genomes were publicly available (via NCBI or WormBase ParaSite WBPS13) [75], and a monogenean outgroup (*Gyrodactylus salaris*, PRJNA244375) [76] were identified. The amino acid sequences inferred from these genes were subjected to automated quality improvement for multiple sequence alignment (AQUA; v.1.1) [77]. In brief, alignments were constructed using the programs MUSCLE (v.3.8.31, RRID:SCR_011812) [78] and MAFFT (v.7.271, RRID:SCR_011811) [79] and then refined using RASCAL (v.1.34) [80]. Alignments with a score of <0.8 (NorMD) [81] were optimized and merged into subsets using the program PartitionFinder (v.2.1.1) [82], removing those that did not contain all 20 amino acids and/or those that represented mitochondrial or viral amino acid replacement matrices. Remaining subsets (*n* = 186) were subjected to analysis using the maximum likelihood (ML) and Bayesian inference (BI) tree-building methods. For ML, analysis of the replacement matrices inferred from each subset in the alignment was conducted using the program RAxML (v.8.2.9, RRID:SCR_006086) [83]. For BI, 4 Markov chains were run for 1,000,000 Markov chain Monte Carlo generations (metropolis-coupled), and trees were sampled every 100 generations using the program MrBayes (v.3.2.6, RRID:SCR_012067) [84], applying the same replacement matrices as used for ML. After the first 25% of trees were discarded as burn-in, Bayesian posterior probabilities were calculated on the basis of the remaining trees; an analysis was completed when the potential scale reduction factor was ≈1 and the average standard deviation of split frequencies was ≈0. Trees were displayed using FigTree (v.1.31, RRID:SCR_008515) [85].

### Improved genome assembly

**Figure 1:**
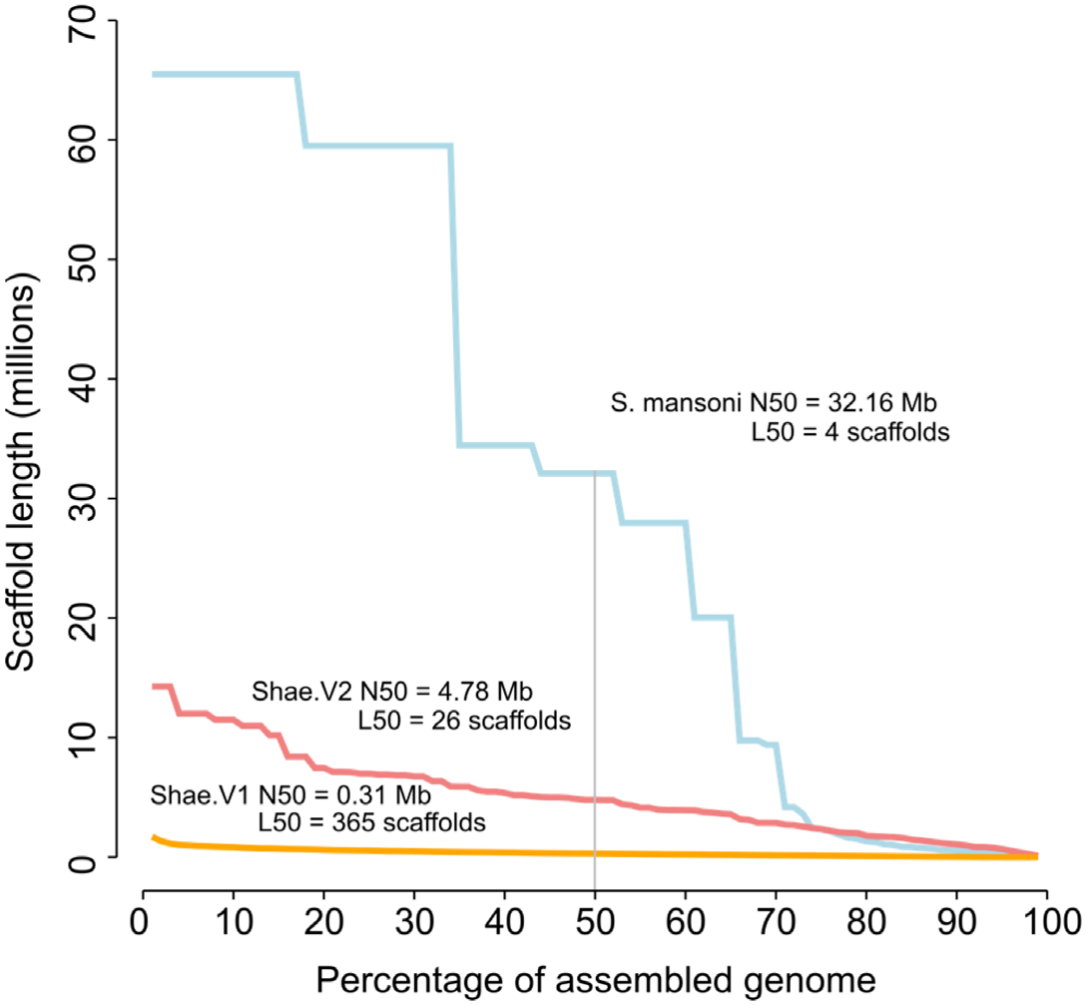
Comparison of schistosome genome assembly quality metrics. Scaffold lengths, N50, and L50 values for *Schistosoma haematobium* genome version 2 (Shae.V2), version 1 (Shae.V1), and *S. mansoni* are shown.

Approximately 3 million error-corrected PacBio reads with an average length of 2,410 nucleotides (nt) were sequenced from ∼33 µg of whole-genome–amplified DNA, achieving 19-fold coverage of the *S. haematobium* genome (Supplementary Table S1). In addition, 350 million reads (95-fold coverage) were sequenced from the Chicago library (Supplementary Table S1). Following filtering, published Illumina mate-pair and short reads (BioProject: PRJNA78265) [14], corrected PacBio reads, and Chicago reads were used to rescaffold and assemble Shae.V1 contigs into the refined Shae.V2 genome for *S. haematobium* (Table 1). The latter genome was assembled into 666 scaffolds (previously 99,953) with a mean length of 557,649 bp (previously 3,853 bp), an N50 of 4.8 Mb (previously 0.31 Mb), and an L50 of 26 scaffolds (previously 365). Approximately 23.42% of the genome assembled into scaffolds of >100,000 bp in length (previously 0.96%), with the longest scaffold containing 14.3 million bp (previously 1.8 million bp) (Table   1 and Fig. 1). In addition, the new assembly was more contiguous, with 15,113 gaps composed of 950,957 ambiguous nucleotides (“Ns”), representing 0.26% (previously 6.02%) of the genome (Table 1).

**Table 1:** Characteristics of the version 2 (Shae.V2) and version 1 (Shae.V1) *Schistosoma haematobium* genomes

Characteristic	Shae.V2	Shae.V1
Number of scaffolds	666	99,953
Total length of all scaffolds	371,394,055	385,110,549
Range of scaffold lengths	518–14,276,808	100–1826,302
Mean scaffold length	557,649	3,853
Median scaffold length	5,586	142
Scaffolds >100 kb* (%)	23.42	0.96
Scaffolds >1 Mb (%)	13.66	0.02
Scaffolds >10 Mb (%)	0.75	0
Scaffold N50	4,779,868	306,738
Scaffold L50	26	365
GC content (excluding Ns)	34.53%	32.19%
Ambiguous bp (Ns)	0.26%	6.02%

^*^Nucleotides.

**Figure 2:**
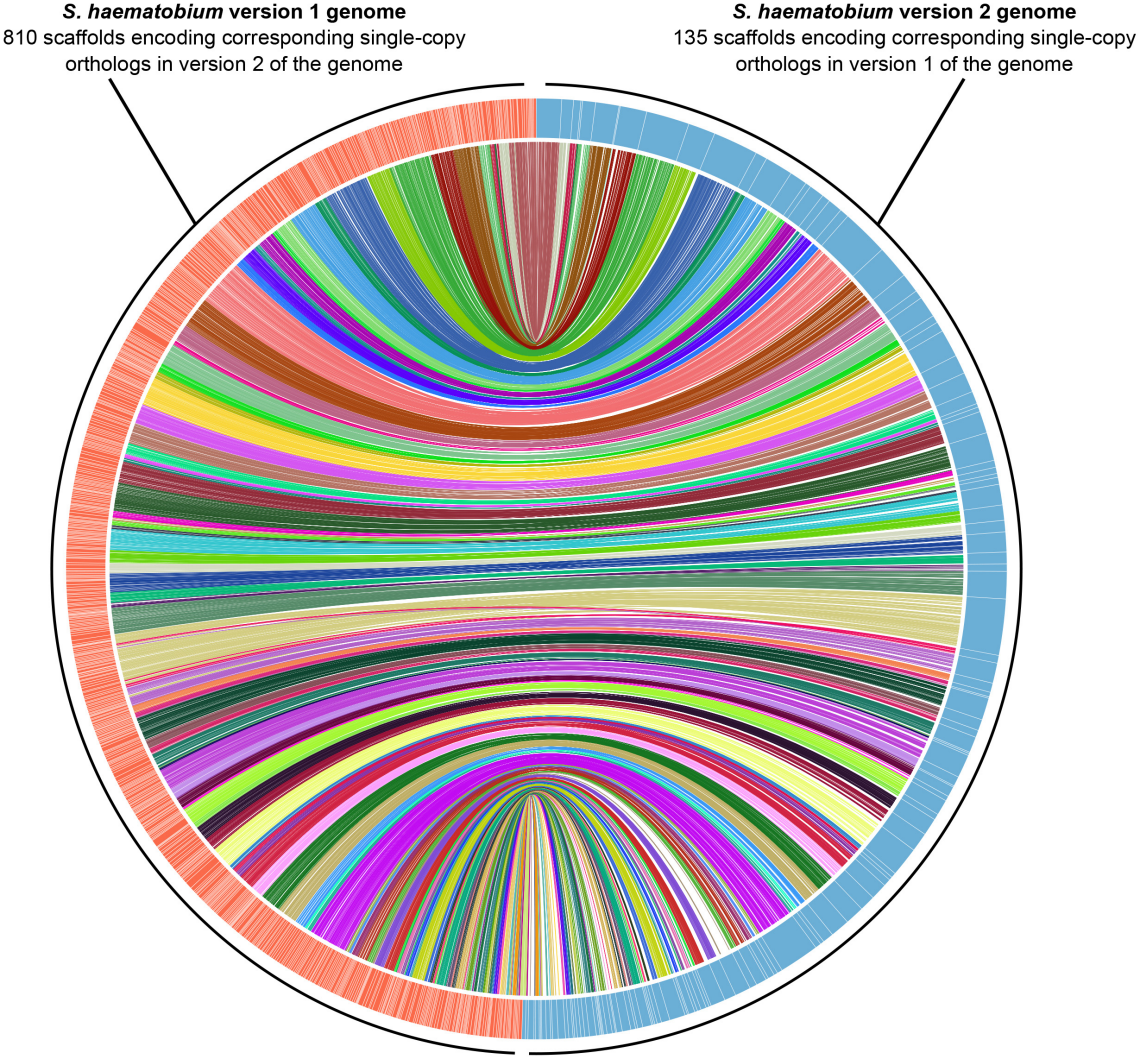
Comparison of the synteny and contiguity of assemblies for *S. haematobium* version 1 (Shae.V1) and version 2 (Shae.V2) genomes. Shae.V1 scaffolds (*n* = 810) are represented by orange bars and are linked with 135 Shae.V2 scaffolds (light blue bars). Scaffolds are arranged as a circular plot based on 5,506 regions containing single-copy orthologs (SCOs, each represented by a line connecting an orange with a blue scaffold). SCO lines have distinct colours for each Shae.V2 scaffold.

**Figure 3:**
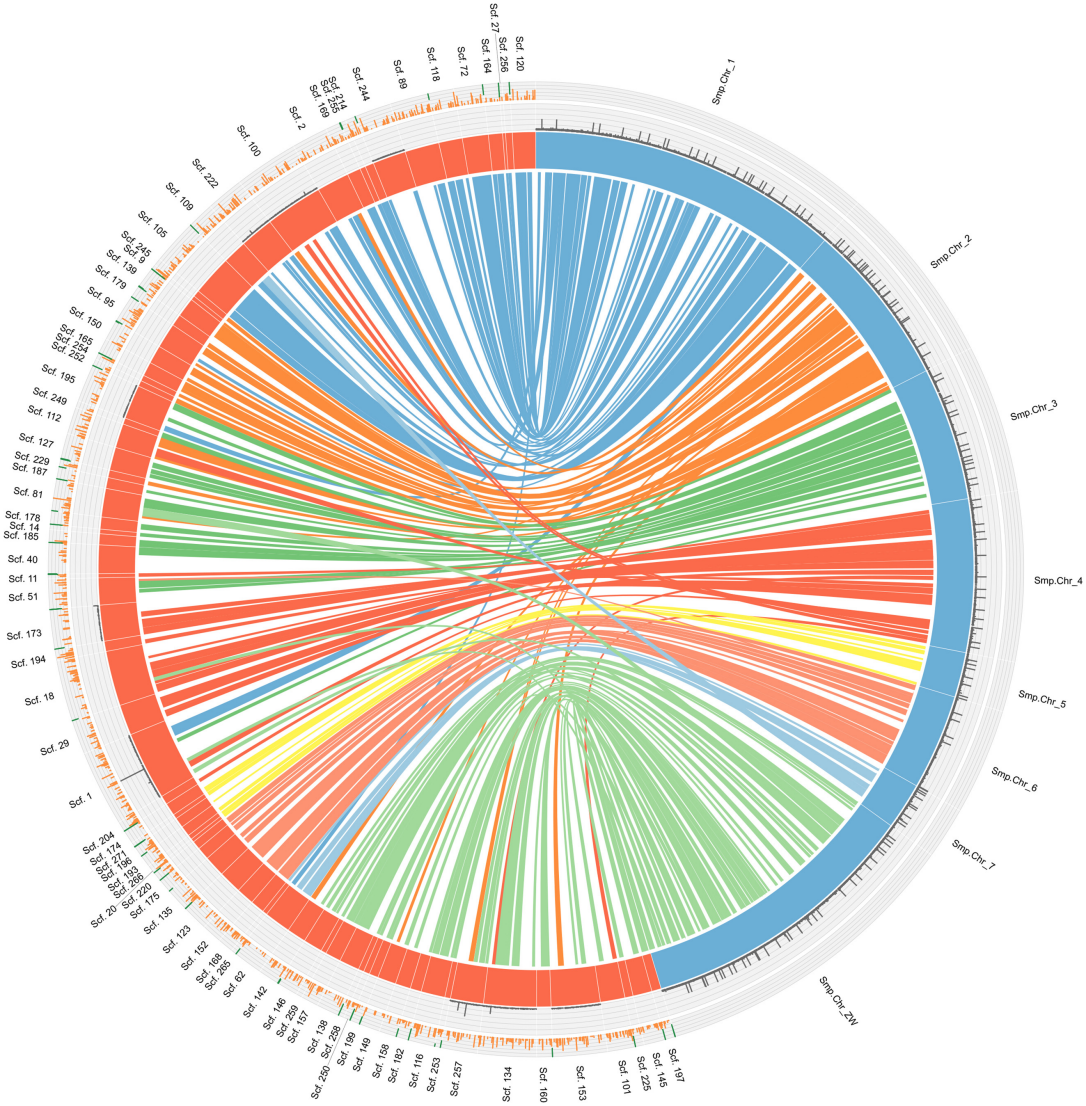
Comparison of the synteny, contiguity, and integrity of assemblies for *S. haematobium* version 2 (Shae.V2) and *S. mansoni* (WBPS8). Shae.V2 scaffolds (*n* = 79) are represented by orange bars and are linked with 8 *S. mansoni* chromosomes (light blue bars). Scaffolds are arranged in a circular plot based on 218 regions containing single-copy orthologs (SCOs, each represented by a line connecting an orange with a blue scaffold). SCO lines have distinct colours for each *S. mansoni* chromosome. Additionally, gaps (“Ns”) are represented as black histograms on a separate track, with the Y-axis representing the size of the region containing ambiguous nucleotides (range, 0–5,013). On the outer track, orange histograms represent areas of >1,000 bp in length for which the coverage of “properly paired” reads was <5 reads. Higher histograms represent longer regions. Dark green histograms within the same track represent regions of low “physical” coverage. The lower the histograms “drop” from the top of the track, the larger is the size of the regions that have “physical” coverage of <10 reads.

A comparison of the Shae.V2 genome to that of Shae.V1 (Fig. 2) or *S. mansoni* (WBPS8) (Fig. 3) inferred 5,506 and 218 syntenic regions containing SCOs, respectively. For *S. mansoni*, all 8 chromosomes comprising 258,697,509 bp (representing 71.0% of the entire *S. mansoni* genome) were represented by a total of 79 *S. haematobium* scaffolds comprising 303,401,942 bp (representing 81.7% of the entire *S. haematobium* genome), confirming a high level of completeness of the Shae.V2 assembly (Fig. 3). For Shae.V1, SCOs linked 135 Shae.V2 scaffolds (total length: 361,192,130 bp, representing 97.3% of the Shae.V2 genome; mean length: 2,675,500 bp) with 810 Shae.V1 scaffolds (total length: 268,521,193 bp, representing 71.4% of the Shae.V1 genome; mean length: 331,508 bp), demonstrating a substantial increase in genome integrity through an ∼6-fold improvement in the contiguity of the new assembly (Fig. 2).

A comparison of the number of gaps in the portion of the Shae.V2 assembly representing the *S. mansoni* chromosomes (Fig. 3) showed that the improved *S. haematobium* assembly contained fewer (*n* = 3,128) gaps than the *S. mansoni* genome assembly (*n* = 5,861), representing a total of 122,623 bp (*S. mansoni*: 1,454,291 bp). Most of the gaps in the Shae.V2 assembly (96.8%) were either 25 bp (*n* = 2,636; introduced by PBJelly) or 100 bp (*n* = 391; introduced by HiRise) long, whereas for *S. mansoni*, 92.5% of them were either 200 bp (*n* = 5,298) or 2,000 bp (*n* = 123) long.

### Refined gene set

Gene models from the Shae.V1 gene set were merged and/or refined and successfully transferred to the Shae.V2 genome by consolidating a total of 37,190 inferred gene models. These models were either predicted by AUGUSTUS (*n* = 2,132), EVM (*n* = 9,633), GENEMARK (*n* = 161), MAKER2 (*n* = 8,310), or SNAP (*n* = 518), or directly inferred from the Shae.V1 gene set by liftOver (*n* = 7,244) or RATT (*n* = 9,192). The final, merged set included 9,314 genes and represented the 11,140 gene models present in the Shae.V1 gene set. In 1,081 cases, ≥2 gene models in Shae.V1 were merged into a single gene model for Shae.V2. In contrast, 76 gene models in Shae.V1 were split into multiple models, representing a total of 178 genes in Shae.V2.

**Figure 4:**
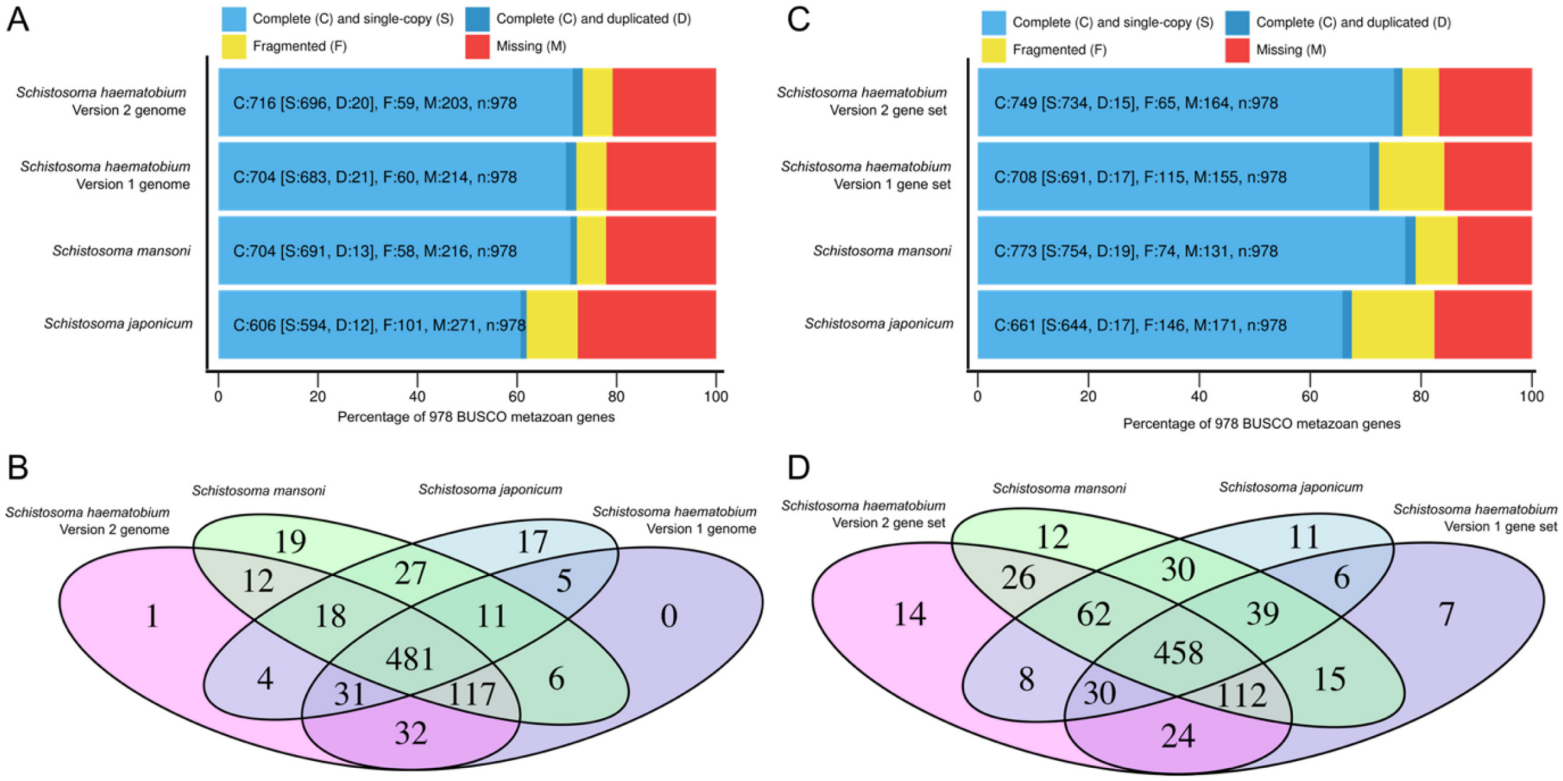
Assessment of genome completeness based on the identification of 978 curated, single-copy, metazoan genes in genomes (A, B) and gene sets (C, D) for schistosomes, using the program BUSCO. The proportion of BUSCO genes identified as complete (single or duplicated), fragmented, or missing (genome mode: A; gene set mode: C) and the number of predicted gene models homologous to complete BUSCO genes (genome mode: B; gene set mode: D) are shown for each genome.

The level of completeness of the Shae.V2 gene set was determined by assessing the presence of 978 BUSCO genes both in the genome (Fig. 4 A and B; Table 2) and in the gene set (Fig. 4C and D; Table 2). For both modes of inference (i.e., genome-based and gene set−based) used in BUSCO, we predicted more complete, single-copy genes and fewer fragmented and missing genes in the Shae.V2 than the Shae.V1 gene set. Comparisons showed that the Shae.V2 gene set was predicted to be nearly as complete as that of *S. mansoni* and substantially more complete than that of *S. japonicum*.

**Table 2:** Assessment of schistosome genome and gene set completeness through the identification of 978 curated, single-copy, metazoan genes (BUSCOs)

Dataset	Complete BUSCOs (%)	Complete and single-copy BUSCOs (%)	Complete and duplicated BUSCOs (%)	Fragmented BUSCOs (%)	Missing BUSCOs (%)
Genome					
*Schistosoma haematobium* version 2	716 (73.22)	696 (71.17)	20 (2.05)	59 (6.04)	203 (20.76)
*Schistosoma haematobium* version 1	704 (71.99)	683 (69.84)	21 (2.15)	60 (6.14)	214 (21.89)
*Schistosoma mansoni*	704 (71.99)	691 (70.66)	13 (1.33)	58 (5.94)	216 (22.09)
*Schistosoma japonicum*	606 (61.97)	594 (60.74)	12 (1.23)	101 (10.33)	271 (27.71)
Gene set					
*Schistosoma haematobium* version 2	749 (76.59)	734 (75.06)	15 (1.54)	65 (6.65)	164 (16.77)
*Schistosoma haematobium* version 1	708 (72.40)	691 (70.66)	17 (1.74)	115 (11.76)	155 (15.85)
*Schistosoma mansoni*	773 (79.04)	754 (77.10)	19 (1.95)	74 (7.57)	131 (13.40)
*Schistosoma japonicum*	661 (67.59)	644 (65.85)	17 (1.74)	146 (14.93)	171 (17.49)

### Phylogenetic position of *S. haematobium* in relation to other parasitic trematodes

**Figure 5:**
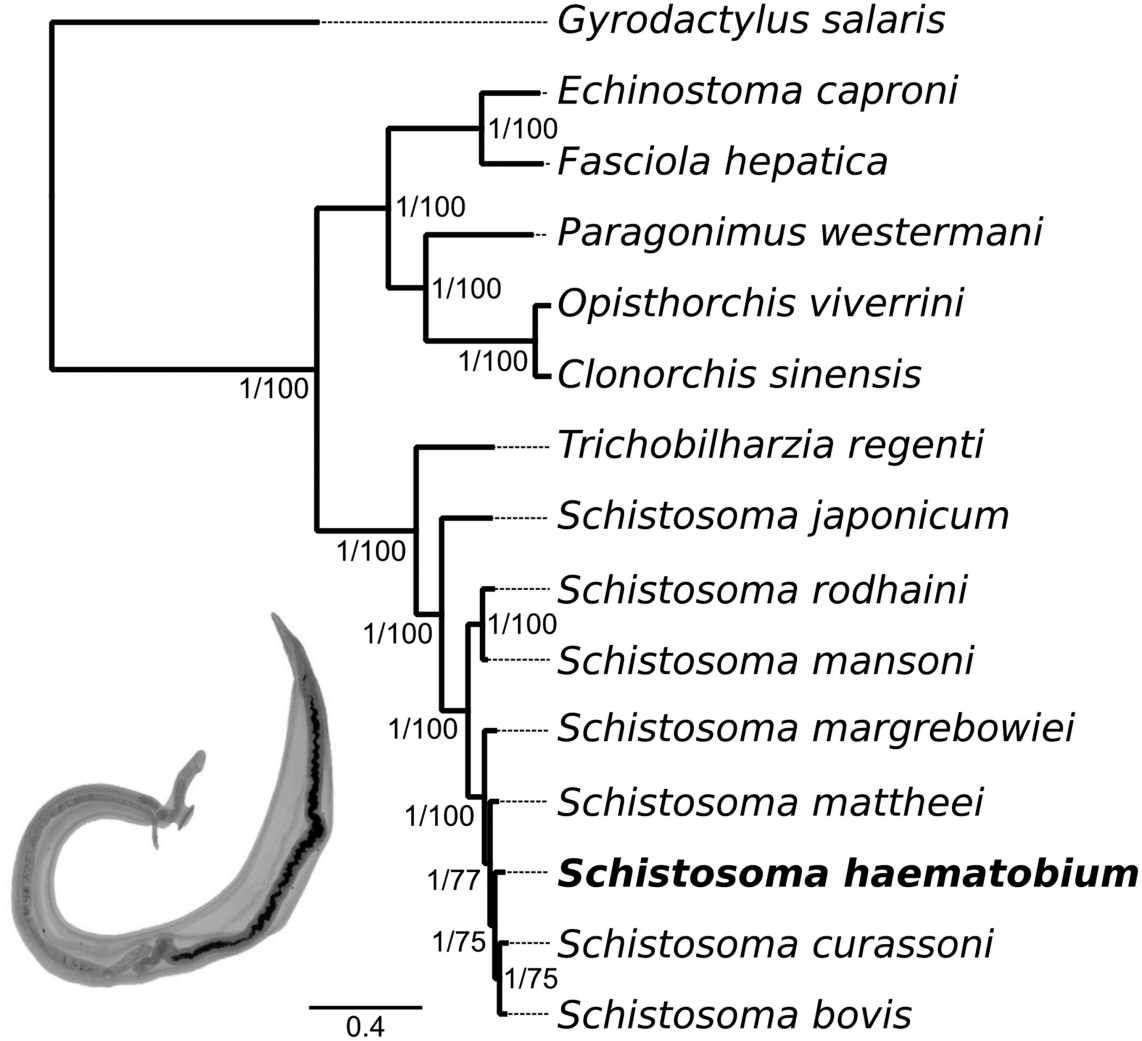
Phylogenetic position of *Schistosoma haematobium* relative to other representatives of the class Trematoda, for which draft genomes were available. Trees constructed using Bayesian inference (BI, shown) and maximum likelihood (ML) analyses of amino acid sequence data inferred from 186 single-copy orthologs (SCOs) had the same topology. Nodal support values for BI and ML analyses are indicated at each branch (posterior probability/bootstrap support). Branch lengths represent the numbers of amino acid substitutions per site at aligned positions. *Gyrodactylus salaris* (class Monogenea) represents the outgroup. Inset image shows a pair of adult schistosomes.

Phylogenetic analysis of concatenated amino acid sequence data inferred from 186 SCOs using BI and ML tree-building methods confirmed the phylogenetic position of *S. haematobium* relative to other representatives of the class Trematoda for which draft genomes were available in public databases (Fig. 5). Clades representing the orders Plagiorchiida (intestinal fluke *E. caproni*, liver fluke *F. hepatica*, and lung fluke *P. westermani*) and Opistorchiida (liver flukes *C. sinensis* and *O. viverrini*) were basal to the family Schistosomatidae (blood flukes) [73, 74]. Within the schistosome clade, *T. regenti* (bird schistosome) was located basal to the genus *Schistosoma*, which was divided into the Asian clade (represented by *S. japonicum*), the *S. mansoni* group (represented by *S. mansoni* and *S. rodhaini*), and the *S. haematobium* group [86]. The 5 representatives of the latter group included here were very closely interrelated, consistent with previous phylogenetic analyses and with the ability of some species to cross-hybridize [73, 86].

## Discussion

Short-read sequencing technologies have enabled the sequencing of genomes for a plethora of organisms, including those of complex eukaryotic pathogens, to a high-quality draft status [14, 29, 42]. Although useful, most draft genomes are fragmented, and substantial efforts are now required to achieve more contiguous assemblies. Recently, long-read technologies have substantially improved our prospects to define accurate genomes for eukaryotic organisms [24, 42, 87, 88]. Here, we harnessed long-read and long-range sequencing, together with existing short-read data, to achieve a substantially enhanced genome assembly for *S. haematobium* that is comparable or even superior to those for related schistosome species (Figs 1 and 3). Because the quality of a genome assembly has a substantial impact on downstream analyses, in particular gene annotation and single-nucleotide polymorphism calling [89, 90], this improved genomic resource will accelerate systems biological research of *S. haematobium* and related schistosomes.

By combining established gene (re-)annotation pipelines [25, 33] and by incorporating evidence from closely related species for which high-quality genomes and gene sets were available, we inferred a gene set that is as complete as that of *S. mansoni*, based on the analysis of conserved SCOs. Importantly, by using a gene transfer approach, instead of re-predicting the complete gene set *de novo*, we retained gene models curated previously for *S. haematobium*, including those coding for key families of proteins, such as kinases [19] and GPCRs [18]. In addition, a synteny analysis using the improved gene set revealed that, overall, there is concordance between the improved assembly for *S. haematobium* and that of *S. mansoni*.

**Figure 6:**
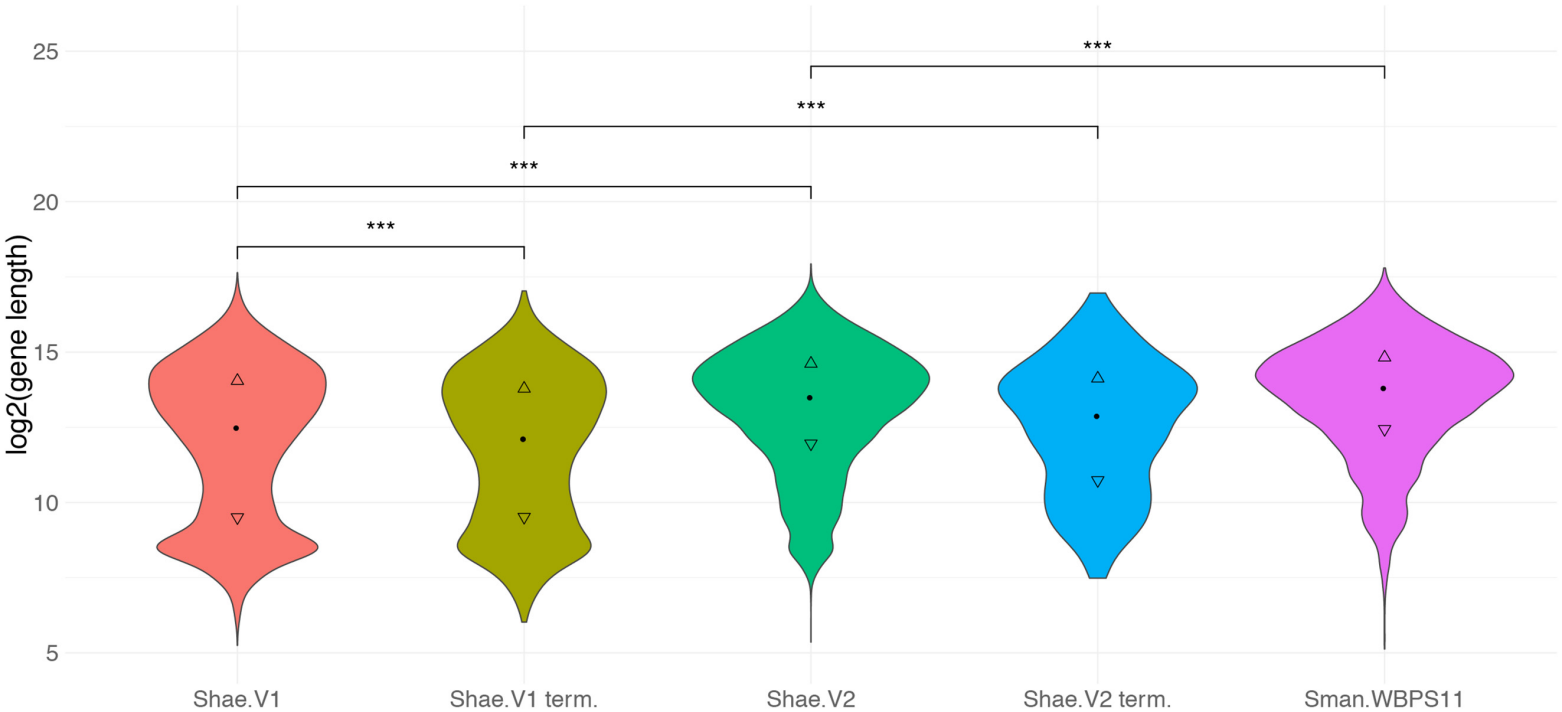
Distribution of gene length for gene sets representing *Schistosoma haematobium* (Shae.V1 and Shae.V2) and *S. mansoni* (Sman.WBPS11). Additionally, distributions are shown for terminal genes (i.e., genes encoded at the start or end of a scaffold) for both *S. haematobium* gene sets (“Shae.V1 term.” and “Shae.V2 term.”). Statistically significant differences among distributions (independent 2-group Mann-Whitney *U* test) are indicated for *P* ≤ 0.001 (***).

Despite this concordance, we identified some differences. For example, the Shae.V2 gene set is ∼8% smaller than that of *S. mansoni* and ∼16% smaller than Shae.V1. The higher number of gene models in Shae.V1 might be explained by a more fragmented assembly, resulting in the prediction of more, incomplete gene models. This proposal is supported by significantly shorter genes (mean: 11,907 bp; median: 5,773 bp) for Shae.V1 compared with Shae.V2 (mean: 18,332 bp; median: 11,759 bp) and by the finding that genes predicted at the start or end of a scaffold were, on average, significantly shorter for Shae.V1 than for Shae.V2 (Fig. 6). The lower number of fragmented BUSCO genes identified in Shae.V2 compared with Shae.V1 lends additional support to this hypothesis. Our findings here are consistent with results for *S. mansoni*, where a substantial improvement [29] of the initial draft genome [91] led to hundreds of merged or discarded gene models and, overall, to a reduced number of predicted genes.

For the most recent *S. mansoni* gene set (WBPS11), both the mean length of genes (21,785 bp) and number of genes (*n* = 10,131) are higher than for Shae.V2, suggesting a more complete assembly and gene set. However, the length distribution of genes is comparable between the 2 species, and contrasts with that for Shae.V1, which shows a clear bias toward shorter genes (Fig. 6). Furthermore, it is plausible that the size of the gene set and the mean gene length for *S. mansoni* are higher than for Shae.V2, because additional RNA-Seq data available for *S. mansoni* (e.g., for the cercarial stage) provided evidence for minimally or selectively expressed transcripts, thus facilitating the detection of novel gene models [26, 29]. In the future, additional RNA-Seq data from multiple developmental stages (including miracidia, sporocysts, and cercariae), for which data are currently unavailable, as well as long-read RNA-Seq data (cf. [92]), should assist in the curation of gene models and the discovery of new transcripts for *S. haematobium*. Another possible reason for a smaller inferred gene set might relate to the gene transfer approach used here [51, 52] that did not include *de novo* prediction of genes in regions that previously did not have gene annotations.

In addition to the observed differences between the 2 most complete schistosome gene sets (*S. mansoni* and now *S. haematobium*), we also detected a number of differences in the associated genome assemblies (Fig. 3). For instance, *S. haematobium* scaffolds that contained gaps (e.g., scaffolds 1, 134, 153, and 257) tended to align to multiple (*n* = 2–6) distinct *S. mansoni* chromosomes, suggesting mis-assemblies. Similarly, there were scaffolds without gaps in the *S. haematobium* assembly (e.g., scaffolds 109, 142, and 149), which corresponded to multiple regions in distinct *S. mansoni* chromosomes that contained gaps, suggesting some incorrect scaffolding in the *S. mansoni* assembly. However, in both cases, it is possible that such regions do differ between the 2 species and are indeed the result of genome rearrangements. Whether these discrepancies represent mis-assemblies or stem from genomic rearrangement events could be the subject of comparative investigations using additional long-read sequencing in the future.

The goal here was to provide a high-quality genomic resource for *S. haematobium*, which will enable in-depth gene (re-)annotation using short- and long-read RNA-Seq data and, more broadly, serve as a reference for functional and population genomics investigations of schistosomes. Overall, despite some differences in gene numbers and scaffold synteny, the BUSCO analysis presented here demonstrated and confirmed a step-change improvement in contiguity for the *S. haematobium* genome assembly and for the gene set, compared with the first draft (Shae.V1). Also, it provided evidence for an assembly quality that is comparable to the best available genome for *S. mansoni* [29]. Achieving a chromosome-contiguous assembly is the ultimate goal, which will provide substantial benefits to the research community and should underpin systems biological investigations and the discovery of new disease interventions.

## Availability of supporting data and materials

The genome assembly and gene set are available from NCBI (BioProject: PRJNA78265), and all associated raw read data are available from the SRA under the accession numbers SRR8485134–SRR8485168. All supporting data and materials are available in the *GigaScience* GigaDB database [93].

## Additional files

**Supplementary Table S1**. Genomic sequence data derived from Chicago and PacBio sequencing libraries of *Schistosoma haematobium*.

giz108_GIGA-D-19-00167_Original_Submission

giz108_GIGA-D-19-00167_Revision_1

giz108_Response_to_Reviewer_Comments_Original_Submission

giz108_Reviewer_1_Report_Original_Submission Rodrigo Baptista, Ph.D. -- 5/24/2019 Reviewed

giz108_Reviewer_1_Report_Revision_1 Rodrigo Baptista, Ph.D. -- 7/18/2019 Reviewed

giz108_Reviewer_2_Report_Original_Submission Krystyna Cwiklinski -- 6/5/2019 Reviewed

giz108_Supplemental_File

## Abbreviations

AIDS: acquired immunodeficiency syndrome; bp: base pair; BI: Bayesian inference; BLASR: basic local alignment with successive refinement; BUSCO: Benchmarking Universal Single-Copy Orthologs; EVM: EVidenceModeler; GAG: Genome Annotation Generator; GFF: general feature format; GPCR: G protein-coupled receptor; HIV: human immunodeficiency virus; kb: kilobase pair; LASTZ: Large-Scale Genome Alignment Tool; MAFFT: Multiple Alignment using Fast Fourier Transform; Mb: megabase pair; ML: maximum likelihood; NCBI: National Center for Biotechnology Information; nt: nucleotide; ORF: open reading frame; PacBio: Pacific Biosciences; PASA: Program to Assemble Spliced Alignments; RATT: Rapid Annotation Transfer Tool; RAxML: Randomized Axelerated Maximum Likelihood; RNA-Seq: RNA sequencing; SCO: single-copy ortholog; SMRT: single-molecule real time; SNAP: Semi-HMM-based Nucleic Acid Parser; SNAP-align: Scalable Nucleotide Alignment Program; SRA: Sequence Read Archive.

## Competing interests

The authors declare that they have no competing interests.

## Funding

Support from the National Health and Medical Research Council (NHMRC) of Australia, the Australian Research Council and Melbourne Water Corporation, The University of Melbourne (BIP) (R.B.G.), and the National Cancer Institute, National Institutes of Health, USA (award R01CA164719) (P.J.B.) is gratefully acknowledged. P.K.K. holds an NHMRC Early Career Research Fellowship. N.D.Y. holds an NHMRC Career Development Fellowship.

## Authors’ contributions

B.W., D.R., P.J.B., R.B.G., and N.D.Y. designed the study and acquired funding. B.W., D.R., and P.J.B. provided material for sequencing through the NIAID Schistosomiasis Resource Center, at the Biomedical Research Institute, Rockville, Maryland, for distribution through BEI Resources, NIH-NIAID Contract HHSN272201000005I. T.M.C., Y.L.L., and K.G.C. carried out PacBio sequencing. A.J.S., P.K.K., and N.D.Y. carried out genome assembly, gene prediction, and all other analyses. A.J.S., R.B.G., and N.D.Y. wrote the manuscript with contributions from all co-authors.
