# Supplementary material for: High-quality Schistosoma haematobium genome achieved by single-molecule and long-range sequencing
Source: Gigascience. 2019 Sep 5;8(9):giz108. doi: 10.1093/gigascience/giz108 (PMC6736295; doi:10.1093/gigascience/giz108)
Supplement: giz108_GIGA-D-19-00167_Revision_1 [file giz108_giga-d-19-00167_revision_1.pdf]

GigaScience

High-quality Schistosoma haematobium genome achieved by single-molecule and long-range sequencing

--Manuscript Draft--

Manuscript Number:	GIGA-D-19-00167R1
Full Title:	High-quality Schistosoma haematobium genome achieved by single-molecule and long-range sequencing
Article Type:	Data Note
Funding Information:	National Cancer Institute, National Institutes of Health, USA (R01CA164719) — Prof Paul Brindley<br>National Health and Medical Research Council (NHMRC) of Australia (1102322) — Prof Robin Gasser<br>Australian Research Council (N/A) — Prof Robin Gasser<br>Melbourne Water Corporation (N/A) — Prof Robin Gasser<br>The University of Melbourne (N/A) — Prof Robin Gasser
Abstract:	Background: Schistosoma haematobium causes urogenital schistosomiasis, a neglected tropical disease affecting > 100 million people worldwide. Chronic infection with this parasitic trematode can lead to urogenital pathology including female genital schistosomiasis (FGS) and bladder cancer. At the molecular level, little is known about the biology of this blood fluke and the pathogenesis of the disease that it causes. To support molecular studies of this carcinogenic worm, we reported a draft genome for S. haematobium in 2012. Although a useful resource, the utility of this draft genome has been somewhat limited by its fragmentation. Findings: Here, we systematically enhanced the draft genome of S. haematobium using a single-molecule and long-range DNA sequencing approach. We achieved a major improvement in the accuracy and contiguity of the genome assembly, making it superior or comparable to assemblies for other schistosome species. We transferred curated gene models to this assembly and, using enhanced gene annotation pipelines, inferred a gene set with as many or more complete gene models compared with those of other well-studied schistosomes. Employing conserved, single-copy orthologs, we assessed the phylogenetic position of S. haematobium in relation to other parasitic flatworms for which draft genomes were available. Conclusions: We report a substantially enhanced genomic resource that represents a solid foundation for molecular research on S. haematobium and is poised to better underpin population and functional genomic investigations, and to accelerate the search for new disease interventions.
Corresponding Author:	Andreas Julius Stroehlein, PhD<br>The University of Melbourne<br>Melbourne, Victoria AUSTRALIA
Corresponding Author Secondary Information:	
Corresponding Author's Institution:	The University of Melbourne
Corresponding Author's Secondary Institution:	
First Author:	Andreas Julius Stroehlein, PhD
First Author Secondary Information:	
Order of Authors:	Andreas Julius Stroehlein, PhD<br>Pasi K Korhonen<br>Teik Min Chong

	Yan Lue Lim
	Kok Gan Chan
	Bonnie Webster
	David Rollinson
	Paul Brindley
	Robin Gasser
	Neil Young
**Order of Authors Secondary Information:**	
**Response to Reviewers:**	Dr Hongling Zhou<br>Junior Editor<br>GigaScience<br><br>25 June 2019<br><br>Dear Dr Zhou,<br><br>RE: GIGA-D-19-00167.R1<br><br>Herewith please find the R1 manuscript entitled "High-quality Schistosoma haematobium genome achieved by single-molecule and long-range sequencing" by Stroehlein et al., submitted for publication in GigaScience.<br><br>We thank you and the two reviewers for their positive and constructive reports on our manuscript. We are pleased that both reviewers are of the opinion that the improved genome assembly represents a very important resource for the research community and that it will serve as a useful reference for future investigations.<br><br>The present rejoinder addresses the reviewers' suggestions and recommendations (responses in bold-type) in a point-by-point manner:<br><br>Reviewer #1:<br><br>1.1. In the Background (line 101-109), the group mentioned the complex nature of its genome (repeats and segmental duplication) that was limiting a proper assembly structure using short reads. Such as S. mansoni, the annotation is also challenging since the gene structure of these worms are different from model organisms, for example, S. mansoni has lots of mini-exons (some with 3-6 nucleotide) with large introns. Without evidence, most of these are missed and not well detected by ab initio approaches. I would recommend adding something talking about this complexity, since the group did also a re-annotation of its genome.<br><br>RESPONSE: We have included a sentence on the challenges of gene prediction in non-model organisms with unique gene structures and have provided relevant references pertaining to schistosomes.<br><br>1.2. Line 184: add scalable nucleotide alignment Program (SNAP-align), Since you also use another tool called SNAP (Semi-HMM-based Nucleic Acid Parser - line 247) for annotation.<br><br>RESPONSE: We thank the reviewer for raising this point. We have amended the text to clarify that these are two distinct programs.<br><br>1.3. Line 199-202: the group was correct to fill gaps using PBJELLY using PacBio reads and scaffolding using SSPACE-LongRead. But there are two steps needed to make sure that the best possible assembly was produced. Long-read technologies unfortunately still have problems in the base call quality and a 19x fold would not be enough to correct all possible errors, so using the already generated Illumina sequence from Shae.V1 the group could fill gaps or extend contigs using Image (PMID: 22678431) and then polishing erroneous base calls using ICORN2 (PMID: 22678431)

or Pilon (PMID: 25409509).

RESPONSE: Reviewer #1 suggests two additional steps to improve assembly using Illumina reads: (i) error-correction of PacBio reads and (ii) gap-filling. We wish to emphasise that both of the suggestions have already been included in our analysis and are described in the Methods section. Therefore, we believe that no additional steps are needed to obtain the best possible assembly. As described in the Methods section, for “pre-assembly processing of sequencing data”, we used published Illumina short-read libraries to error-correct the PacBio reads (referred to as “corrected PacBio reads” later on in the manuscript), employing the program LoRDEC (v.0.3). In addition, we have carried out steps to improve scaffolding and to close gaps using the same Illumina data sets employing Haplomerger 2 (v.3.2), as described in “Genome Assembly (2)”. We have amended the text of these sections to further clarify the error-correction process.

1.4. Line 221-277: As I mentioned above, this genome has an intriguing annotation, S. mansoni for example is being revisited all the time and just now using IsoSeq from PacBio, they are finally finding new evidences for mini-exons and isoforms on its genome annotation. I didn't see any phrase specifying if this was done (using Artemis or Webapollo), where you can indeed check if you have enough support from different resources of this gene. I'm concerned of not doing this and just keeping the "longest ORF" since Schistosoma biology does not necessarily follow this assumption.

RESPONSE: We agree with Reviewer #1’s opinion that schistosomes have an intriguing genome and are aware of the many fascinating characteristics yet to be explored. However, as stated in the Discussion section, the goal of the present study was to provide a high-quality genomic resource for S. haematobium to enable in-depth gene (re-)annotation, employing short- and long-read RNA-Seq data sets in the near future. In this context, a detailed re-annotation of the entire genome was beyond the scope of the present study. The gene annotation inferred here was used principally to evaluate the quality of the assembly, genome synteny and for comparative analyses. In our experience, using currently available short-read RNA-Seq data does not allow transcript isoforms to be accurately inferred, but reveals similar isoforms that differ only by UTR length, instead of accurately resolving alternative usage of internal exons. Detailed re-annotation using long-read RNA sequencing, such as PacBio IsoSeq and/or Oxford Nanopore Direct RNA sequencing, should allow us to resolve many of these features in the future, using the high-quality genomic resources presented here.

1.5. Line 265-277: When keeping the "longest ORF" you mean the whole transcript (all exons). If yes, maybe you need to change this to transcript, if not, I'm a bit worried if doing this is the best approach to get the right answer to your problem (see comment above talking about mini-exons).

RESPONSE: We have elected to use the longest open-reading frame as the representative coding region of a gene, because the objective here was to accurately capture the protein-coding regions and their boundaries for all genes, rather than resolving all possible combinations of exons (transcript isoforms). This aspect is still challenging and inaccurate due to the lack of long-read RNA-Seq data. Technologies producing such data have only recently become available and have improved enough in accuracy to facilitate such analyses in the near future. We thank the reviewer for their suggestion and agree that such technologies should be used in future re-annotation efforts of the enhanced genome of S. haematobium.

1.6. Line 299-309: Which substitution model was used for the ML tree reconstruction? Add this information and the analysis of ModelTest (which predicts the best substitution model should be used in your ML/BI analysis).

RESPONSE: We have used PartitionFinder (v.2.1.1) to establish the best substitution models and associated replacement matrices for each partition, as described in the Methods section. The configuration files (raxml.txt and mrbayes.txt) for the ML and BI trees included in the GigaDB repository linked to the manuscript and contain all of the requested information.

1.7. Line 341-342: the 25 and 100 length sizes gaps are unknown gaps generated by

PBJelly and HiRIse. PBJelly add 25 N bases when it can't be filled and HiRise follows the 100 bp gap window as requested by the community for unknown gap sizes. Usually you need to say this during the process of submission to NCBI, where all 100 bp N sites are considered unknown and other sizes are supported by physical mapping. This needs to be fixed. If you have any physical mapping supporting these gap sizes this needs to be better described in the manuscript (maybe with a supplementary figure).

RESPONSE: These gap sizes of 25 bp and 100 bp are introduced by the programs PBJelly and HiRise, respectively, as pointed out by the reviewer. We have added this information. Gap sizes of > 100 bp are estimated based on paired-end read mapping. Thus, we have set the "minimum number of N's in a row that represents a gap of estimated length" to 101 for the genome submission to NCBI. Although we cannot provide estimates for the unknown gap sizes introduced by PBJelly and HiRise, our coverage analysis (methods described in the last paragraph of the "Genome Assembly" section) showed that scaffolds containing gaps have a 'physical' coverage of ≥ 10 reads (i.e. spanned by ≥ 10 paired-end reads) (Figure 3), thus supporting correct scaffolding.

1.8. Line 125: add the name of the strain or isolate (e.g. MP2012). This helps other research groups in text mining analysis

RESPONSE: There is no name for the strain other than the description that we have provided. However, we have created individual BioSample IDs for the worms isolated in 2012 (used for sequencing for the first draft of the genome, designated MP2012) as well as those isolated and used for PacBio sequencing in the present manuscript (designated MP2018). We have amended the text and included the isolate and BioSample IDs to reflect the origin of those two samples.

1.9. Line 153: add version of the SMRT analysis software used for assembly (SMRTlink is the most recent)

RESPONSE: We have added the version information for the SMRT analysis software.

1.10. Line 123-309: Add versions to all software used, some like samtools (line 190) are missing.

RESPONSE: We have added all missing version information.

1.11. Line 288-292: Add a supplementary table with information about these parasites (with accession number) to help to know which version was used.

RESPONSE: Sequence data was obtained from WormBase ParaSite (WBPS13) or NCBI (where data had not been uploaded to WormBase). We have added this information and accession numbers for all parasite genome data sets used for the construction of phylogenetic trees.

1.12. Line 304: "1,000 bootstrap generations";

RESPONSE: This sentence describes the Bayesian inference analysis, which employs Markov Chain Monte Carlo sampling and not 'bootstrapping' employed by Maximum Likelihood-based tree-building methods (see PMID: 12598693). The support values for the BI analysis are not 'bootstrap support' values, but rather posterior probability (BPP) values. Thus, there is no need to alter the text in the manuscript.

1.13. Whole manuscript: fix inconsistencies: numbers, sometimes 1000 some times 1,000. BP, KB, MB and GB or nt. I recommend the first one.

RESPONSE: We have amended the manuscript accordingly. For numbers of ≥ 10,000, we have used a 'thousands delimiter' to improve readability of large numbers.

1.14. Suggestion for future work: Try to use Hi-C in a future re-assembly project, their sizes are much bigger than Chicago and will help to improve even more your results.

RESPONSE: We thank the reviewer for this suggestion. We will continue to adopt new

Powered by Editorial Manager® and ProduXion Manager® from Aries Systems Corporation

	state-of-the-art sequencing technologies to further improve the genome assembly and annotation in the future.<br><br>Reviewer #2:<br><br>2.1. Although discussed throughout the main body of the manuscript, further emphasis is required in the abstract to reflect that the sequencing carried out as part of this study was not used to assemble a wholly new version of the genome but instead was used to improve the version 1 assembly. The gene model mapping should also be included.<br><br>RESPONSE: The Abstract clearly summarises previous work done to obtain a first draft genome and mentions its limitations. This description is followed by “Here, we systematically enhanced the draft genome of S. haematobium…”. In our opinion, this sentence reflects the fact that the present study reports an improved genome, which is based on a published draft genome and new data sets created in this body of work. Therefore, we see no reason to alter the Abstract. Regarding “gene model mapping”, we have amended a sentence in the Abstract to indicate that the gene models were both transferred from the original gene set and predicted using improved gene annotation pipelines.<br><br>2.2. Line 182: can the authors provide further clarification regarding the unscaffolded contigs used in the Dovetail HiRise pipeline - were these the contigs used for the version 1 scaffold assembly or the contigs that did not scaffold in the original version?<br><br>RESPONSE: The un-scaffolded contigs were the contigs assembled for Shae.V1, before scaffolding. We have amended this sentence to clarify this aspect.<br><br>2.3. Lines 288-292: where are the genomes publicly available? If WormBase ParaSite, mention which version of the genomes were used for the phylogenetic analysis.<br><br>RESPONSE: Genome data were obtained from WormBase ParaSite WBPS13 and NCBI. We have clarified this aspect in the manuscript.<br><br>______________________________<br><br>CONCLUDING REMARKS<br>We thank all reviewers for their time, effort and reports, and you for the time that you have spent handling and appraising the manuscript. We have revised the manuscript to address all of the reviewers’ comments (changes in the revised (R1) manuscript are highlighted in yellow). The suggested changes and recommendations have helped us in our revision. In our opinion, the revised manuscript now meets the standard for publication in GigaScience.<br><br>Yours sincerely,<br><br>Neil D. Young PhD<br>NHMRC Career Development Fellow<br>Faculty of Veterinary and Agricultural Sciences, The University of Melbourne | Corner Flemington Road & Park Drive, Parkville, Victoria 3010, Australia | nyoung@unimelb.edu.au
**Additional Information:**	
**Question**	**Response**
Are you submitting this manuscript to a special series or article collection?	No
**Experimental design and statistics**<br><br>Full details of the experimental design and statistical methods used should be given in the Methods section, as detailed in our	Yes

Minimum Standards Reporting Checklist. Information essential to interpreting the data presented should be made available in the figure legends.<br><br>Have you included all the information requested in your manuscript?	
**Resources**<br><br>A description of all resources used, including antibodies, cell lines, animals and software tools, with enough information to allow them to be uniquely identified, should be included in the Methods section. Authors are strongly encouraged to cite Research Resource Identifiers (RRIDs) for antibodies, model organisms and tools, where possible.<br><br>Have you included the information requested as detailed in our Minimum Standards Reporting Checklist?	Yes
**Availability of data and materials**<br><br>All datasets and code on which the conclusions of the paper rely must be either included in your submission or deposited in publicly available repositories (where available and ethically appropriate), referencing such data using a unique identifier in the references and in the "Availability of Data and Materials" section of your manuscript.<br><br>Have you have met the above requirement as detailed in our Minimum Standards Reporting Checklist?	Yes

Powered by Editorial Manager® and ProduXion Manager® from Aries Systems Corporation

Manuscript
Click here to view linked References
Click here to access/download;Manuscript;167-Stroehlein_ShV2_R1_submit--HL.docx

DATA NOTE

High-quality *Schistosoma haematobium* genome achieved by single-molecule and long-range sequencing

Andreas J. Stroehlein[1], Pasi K. Korhonen[1], Teik Min Chong[2], Yan Lue Lim[2], Kok Gan Chan[2], Bonnie Webster[3], David Rollinson[3], Paul J. Brindley[4], Robin B. Gasser[1,*] and Neil D. Young[1,*]

[1]Department of Veterinary Biosciences, Melbourne Veterinary School, Faculty of Veterinary and Agricultural Sciences, The University of Melbourne, Parkville, Victoria 3010, Australia
[2]Institute of Biological Sciences, Faculty of Science, University of Malaya, 50603 Kuala Lumpur, Malaysia
[3]Parasites and Vectors Division, The Natural History Museum, London SW7 5BD, United Kingdom
[4]School of Medicine & Health Sciences, Department of Microbiology, Immunology & Tropical Medicine, The George Washington University, Washington, DC 20037, United States of America

Email addresses

Andreas J. Stroehlein:	astroehlein@unimelb.edu.au
Pasi K. Korhonen:	pasi.korhonen@unimelb.edu.au
Teik Min Chong	cluster1986@hotmail.com
Yan Lue Lim:	yanluelim@hotmail.my
Kok Gan Chan:	kokgan@um.edu.my
Bonnie Webster:	b.webster@nhm.ac.uk
David Rollinson:	d.rollinson@nhm.ac.uk
Paul J. Brindley:	pbrindley@gwu.edu
Robin B. Gasser:	robinbg@unimelb.edu.au
Neil D. Young:	ndyoung@unimelb.edu.au

[*]**Correspondence address.** Department of Veterinary Biosciences, Melbourne Veterinary School, Faculty of Veterinary and Agricultural Sciences, The University of Melbourne, Parkville, Victoria 3010, Australia. Tel: +61 97312283; Fax: +61 97312000. Email: robinbg@unimelb.edu.au or ndyoung@unimelb.edu.au

ORCID IDs. Andreas J. Stroehlein: 0000-0001-9432-9816; Pasi K. Korhonen: 0000-0002-9957-4674; Kok Gan Chan: 0000-0002-1883-1115; Bonnie Webster: 0000-0003-0930-9314; David Rollinson: 0000-0003-1999-1716; Paul J. Brindley: 0000-0003-1765-0002; Robin B. Gasser: 0000-0002-4423-1690; Neil D. Young: 0000-0001-8756-229X

Abstract

Background: *Schistosoma haematobium* causes urogenital schistosomiasis, a neglected tropical disease affecting > 100 million people worldwide. Chronic infection with this parasitic trematode can lead to urogenital pathology including female genital schistosomiasis (FGS) and bladder cancer. At the molecular level, little is known about the biology of this blood fluke and the pathogenesis of the disease that it causes. To support molecular studies of this carcinogenic worm, we reported a draft genome for *S. haematobium* in 2012. Although a useful resource, the utility of this draft genome has been somewhat limited by its fragmentation. **Findings:** Here, we systematically enhanced the draft genome of *S. haematobium* using a single-molecule and long-range DNA sequencing approach. We achieved a major improvement in the accuracy and contiguity of the genome assembly, making it superior or comparable to assemblies for other schistosome species. We transferred curated gene models to this assembly and, using enhanced gene annotation pipelines, inferred a gene set with as many or more complete gene models compared with those of other well-studied schistosomes. Employing conserved, single-copy orthologs, we assessed the phylogenetic position of *S. haematobium* in relation to other parasitic flatworms for which draft genomes were available. **Conclusions:** We report a substantially enhanced genomic resource that represents a solid foundation for molecular research on *S. haematobium* and is poised to better underpin population and functional genomic investigations, and to accelerate the search for new disease interventions.

Keywords: *Schistosoma haematobium*; genome assembly; single-molecule and long-range sequencing

Background

Human schistosomiasis is a chronic, neglected tropical disease affecting > 200 million people worldwide and resulting in > 300,000 deaths each year [1]. *Schistosoma haematobium* (mainly in Africa; Fig. 1), *S. mansoni* (mainly in Africa and South America) and *S. japonicum* (in Asia) are the three main blood flukes (schistosomes) of humans; the first causes urogenital schistosomiasis, and the other two cause hepatointestinal disease. Urogenital schistosomiasis results principally from a chronic (granulomatous) inflammatory process [2] directed at schistosome eggs entrapped in tissues [2-4], and is accompanied by increased risk for HIV/AIDS and infertility in women [5,6] and for squamous cell carcinoma of the urinary bladder [7]. Despite efforts to control schistosomiasis, it remains endemic in many subtropical and tropical regions of the world. As there is no effective vaccine to protect humans [8], control currently relies heavily on targeted or mass treatment with the drug praziquantel [9], a reliance that risks the emergence of resistance to this compound [9]. In addition, treatment alone does not prevent reinfection. Thus, new, complementary interventions need to be established and implemented in the event that resistance to praziquantel becomes widespread [10], and to underpin efforts to eliminate the disease. Clearly, the development of interventions would be facilitated by sound knowledge and understanding of schistosome biology and the pathogenesis of the disease at the molecular level. However, fundamental and applied research on schistosomes has been neglected, particularly for *S. haematobium* [11], in spite of its high prevalence (> 110 million people) in Africa. Since the London Declaration in 2012 [12], there has been an increased resolve by the scientific and philanthropic communities to tackle this problem [10].

In 2012, we reported a draft nuclear genome of *S. haematobium* (Egyptian strain, maintained at the Biomedical Research Institute, Rockville, Maryland [13]; NCBI:txid6185), assembled from short-read Illumina data derived from a single pair of adult worms [14]. This genome assembly enabled the inference of protein-encoding genes, functional annotation including gene ontology networks and metabolic pathways, and the exploration of the nature and extent of transposable elements [14]. Importantly, it also facilitated systematic comparative studies of genomes and gene families in human blood flukes [14-19]. In conjunction with other developments, including the establishment of a rodent model to study the pathogenesis for *S. haematobium* egg-induced disease [20], and knowledge that *S. haematobium* has a functional RNA interference (RNAi) pathway [21], the first draft genome for *S. haematobium* [14] has underpinned molecular investigations of schistosome biology, urogenital schistosomiasis [22] and associated cancer [23].

Despite the value of this resource for the schistosome research community, the utility of the draft genome assembly has been somewhat compromised by its fragmentation; the first assembly of the genome (designated Shae.V1) consisted of 99,953 contiguous sequences (i.e. scaffolds) that were interrupted by 29,422 gaps. Thus, the order and orientation of many segments of the genome could not be established. Genome finishing of large eukaryotic genomes using short-read sequence data is technically challenging, mostly due to difficulties assembling complex regions that are replete with dispersed repeats and large segmental duplications, which greatly complicates the determination of genome structure and sequence [24-26]. Subsequent annotation can be challenging due to complex and non-canonical gene structures [27]. In addition, gene prediction pipelines trained using data from model organisms are not accurate for divergent species [28]. Nevertheless, recently, advances in sequencing technologies have resulted in a systematic refinement of eukaryotic parasite genomes, enhanced gene sets and an improved understanding of genomic architecture [29-31]. To complement these efforts and to provide an improved foundation for molecular research on *S. haematobium*, herein, we systematically improved the draft genome of *S. haematobium* by

using a combination of single-molecule sequencing technology (PacBio) [32], long-range ('Chicago') library construction and Illumina sequencing, supported by existing Illumina short-read data [14]. Based on this enhanced reference, we refined the gene annotation, by transferring curated gene models from the original assembly and by employing established gene (re-)annotation pipelines [25, 33] and published RNA-seq data [14]. Subsequently, we re-assessed the phylogenetic position of *S. haematobium* relative to other trematodes, for which draft genomes were publicly available, using amino acid sequence data sets inferred from single-copy orthologs shared among all taxa included in the analysis.

Data Description

Sample procurement, preparation and storage

All samples originated from the same Egyptian strain of *S. haematobium* that was used to assemble the first draft genome of *S. haematobium* [14]. This strain is maintained at the Biomedical Research Institute, Rockville, Maryland [13] in *Bulinus truncatus* (intermediate snail host) and *Mesocricetus auratus* (hamster; mammalian definitive host). Hamsters were each infected with 1000 cercariae. Ninety days later, paired adults of *S. haematobium* were collected from *M. auratus*, following the perfusion of the mesenteric and intestinal vessels using physiological saline (37 °C). Worms were prepared and stored as described [14].

Single-molecule and long-range library construction and genomic sequencing

For long-read sequencing (PacBio), genomic DNA (~ 1 μg) was isolated from a single pair of adult worms (i.e. male and female *in copula*; isolate MP2018; BioSample ID: SAMN10797288) of *S. haematobium* using a kit (Chemagic DNA Tissue Extraction Kit, Chemagen), and 25 ng were subjected to whole genome amplification (WGA) using a REPLI-g Single Cell Kit (Qiagen). The amplified DNA was purified and concentrated using 0.45-fold volume of Agencourt AMPure XP magnetic beads (Beckman Coulter Inc.). DNA amount was determined using a Qubit fluorometer dsDNA HS Kit (Life Technologies), and its integrity was verified by agarose gel electrophoresis. WGA DNA (8 μg) was sheared to ~ 10 kb using a g-TUBE (Covaris), purified and concentrated using 0.45-fold volume of washed Agencourt AMPure XP magnetic beads (Beckman Coulter) and examined using an Agilent 2100 Bioanalyzer (Agilent Technologies). This sheared DNA was used to construct a SMRTbell library (~ 2.7 kb average size) employing the SMRTbell Template Preparation Kit (v.1.0; Pacific Biosciences). In brief, sheared DNA was subjected to end-repair, ligation of adaptors and exonuclease digestion of incomplete SMRTbell templates. Thereafter, library sequencing primers were annealed (0.83 nM final concentration) to the SMRTbell template, allowing the P4 DNA polymerase (DNA Polymerase Binding Reagent Kit; Pacific Biosciences) to bind. This complex was immobilized on Magbeads (Pacific Biosciences) using protocols for enhanced loading efficiency. Sequencing was performed on the PacBio RS II system (Pacific Biosciences) using 33 SMRT cells and Sequencing Reagent 2.0 (Pacific Biosciences). Sequence data were collected using a 180-minute movie length and the stage-start option. Adapters, short reads (< 50 bases) and nucleotides with an estimated polymerase read quality value of < 0.75 were removed from the acquired data using the SMRT analysis software (v.2.1.0.0.127824; Pacific Biosciences).

To construct a long-range ('Chicago') linking library, used for scaffolding [34], genomic DNA was isolated from ~ 20 pairs of adult worms (i.e. male and female *in copula*; BioSample ID: SAMN10797287) of *S. haematobium* using a Chemagic DNA Tissue Extraction Kit. In brief, genomic DNA (2 μg) was fragmented to produce 500 ng of high-molecular-weight DNA (mean fragment size: 50 kb), which was reconstituted into chromatin *in vitro* and fixed with formaldehyde. Fixed chromatin was digested with *Dpn*II, the 5'-overhangs were filled in with

biotinylated nucleotides, and the free blunt ends were ligated. After ligation, cross-links were reversed, and the DNA was purified from protein. Purified DNA was treated to remove biotin that was not internal to ligated fragments. The DNA was sheared to a mean fragment size of ~ 350 bp, and sequencing libraries were constructed using NEBNext Ultra enzymes and Illumina-compatible adapters. Biotin-containing fragments were isolated using streptavidin beads before PCR-based enrichment of the library. This library was sequenced (100 bp, paired-end reads) using an Illumina HiSeq 2500 platform following the rapid-run protocol.

Pre-assembly processing of sequence data

First, SMRTbell adapters were removed from PacBio reads using BBMAP (v.37.33, BBmap, RRID:SCR_016965) [35]. Due to a 19-fold coverage, these reads were error-corrected using the program LoRDEC (v.0.3, LoRDEC, RRID:SCR_015814; *correct* and *trim* options) [36]. Second, using existing Illumina paired-end, short-insert libraries (170 bp, 500 bp and 800 bp; NCBI BioProject accession number: PRJNA78265) [14], a *de Bruijn* graph with *k*-mers of length 21 was generated. Third, low-quality bases (Phred quality score < 25), adapters and reads of < 40 nucleotides in length were removed from long-range sequence data, using the program Trimmomatic (v.0.32, Trimmomatic, RRID:SCR_011848) [37].

Genome assembly

The new genome scaffolds (designated Shae.V2 genome scaffolds) were assembled in a stepwise manner:

(1) All contigs comprising the Shae.V1 assembly (*n* = 129,375) [14] were scaffolded using long-range, paired-read data employing the Dovetail HiRise pipeline (v.2.0.5) [34]. In brief, reads were aligned to contigs using the Scalable Nucleotide Alignment Program (SNAP-align; v.1.0dev.67_as) [38], masking out bases that follow a junction of two sites of recognition for the restriction enzyme *Mbo*I (GATCGATC), and removing the penalty assigned to the map quality for any two reads that formed a pair but mapped to different scaffolds. To identify repetitive genomic regions, 500 bp reads from a previous study [14] were aligned to the Shae.V1 contigs using SNAP-align. All alignment files were compressed into the BAM format, sorted and indexed using the program SAMtools (v.1.6-7-g35457e2, SAMTOOLS, RRID:SCR_002105) [39]. Duplicates were removed using the sorted BAM files and Picard tools (v.1.123) [40]. Subsequently, the HiRise pipeline was used to iteratively identify and break mis-assemblies and re-scaffold contigs using an established method [34].

(2) The Haplomerger2 pipeline (v.3.2) [41] was employed to remove redundancy in scaffolds of > 250 bp in length that were generated by HiRise, to improve scaffolding using published 2 kb, 5 kb and 10 kb Illumina mate-pair libraries [14] and to close gaps in scaffolds using published 170 bp, 500 bp and 800 bp paired-end, short-read libraries [14].

(3) Corrected PacBio reads were used to close gaps in scaffolds using PBJELLY2 (PBSuite v.14.9.9) [42].

(4) Corrected PacBio long-read data were also used to improve the assembly of scaffolds using SSPACE-LongRead (v.1.1) [43], requiring three links between scaffolds.

Following assembly, ‘contaminant’ scaffolds with homology to bacteria but without nucleotide sequence homology to schistosome scaffolds were identified by searching the NCBI nt database [44] using BLASTn (v.2.5.1+, BLASTN, RRID:SCR_001598) [45], and removed. The completeness of the Shae.V2 genome assembly was assessed using BUSCO (“Benchmarking Universal Single-Copy Orthologs”; v.3.0, BUSCO, RRID:SCR_015008) [46]

in the genome mode, and compared with BUSCO results for the published Shae.V1, *S. japonicum* and *S. mansoni* assemblies [14, 29, 47]. The lengths and locations of ambiguous nucleotide homopolymer gaps were assessed in each set of genome scaffolds using SeqKit (v.0.6.0) [48]. The coverage of individual Shae.V2 genome scaffolds was assessed by mapping short-insert (insert size: 170 bp and 500 bp), mate-pair (800 bp, 2 kb, 5 kb and 10 kb), Chicago long-range and PacBio reads to the assembled scaffolds using SNAP-align (for Illumina reads) or BLASR (for PacBio reads; v.2.2.0.133377, BLASR, RRID:SCR_000764) [49]. Alignment results were filtered for 'properly mapped pairs' (using *samtools view*, -f2 option) and then stored and sorted in the BAM format. Sorted BAM files were merged, and coverage was determined using *samtools depth* (read coverage) and *bamCoverage* (v.3.0.1; -e option; 'physical' coverage, considering regions spanned by paired-end reads as covered) [50], respectively. Regions of > 1000 nucleotides were designated as "regions of low coverage" if < 5 reads (for read coverage) or < 10 reads (for 'physical' coverage) mapped.

Transfer of existing gene models to newly assembled scaffolds and prediction of a final gene set

Existing protein-encoding gene models for the Shae.V1 gene set [14], stored in the general feature (GFF) format, were transferred to the Shae.V2 scaffolds using liftOver (kentUtils v.302) [51] and the Rapid Annotation Transfer Tool (RATT; v.0.95) [52]. The Shae.V1 gene set included manually or semi-automatically curated gene models published in earlier studies, including those for G-coupled protein receptors (GPCRs) [18], protein kinases [19], annexins [16] and SCP/TAPS [15]. For liftOver, an available repeat library [14] was used to soft-mask both Shae.V1 and Shae.V2 scaffolds using RepeatMasker (v.4.0.5, RepeatMasker, RRID:SCR_012954) [53]. LASTZ (v.1.02.00) [54] and chainNet tools (jksrc20100603 within Haplomerger2 v.3.2) [55] were used to identify aligned 'blocks' in each set of scaffolds. Within aligned blocks, genes from the Shae.V1 gene set were transferred to respective Shae.V2 scaffolds using liftOver. In addition, soft-masked Shae.V1 and Shae.V2 scaffolds and the Shae.V1 gene set were used to transfer protein-encoding gene models to Shae.V2 using RATT. Transferred gene models were stored in the GFF format for further processing.

A final Shae.V2 gene set was inferred by combining gene models transferred from Shae.V1, gene model-evidence derived from transcriptomic data (RNA-Seq), *ab initio* gene predictions, and evidence of genomic regions encoding proteins homologous to predicted proteins in other flatworms using the programs MAKER2 (v.2.3.8) [56] and Evidence Modeler (EVM v.1.1.1) [57] in a stepwise manner:

(1) Available RNA-Seq data for adult (male and female) and egg stages [14] was assembled *de novo* using Trinity (v.2.2.0, Trinity, RRID:SCR_013048) [58]. Assembled, non-redundant, full-length transcripts were predicted using TransDecoder (v.2.1.0) [59].
(2) Available RNA-Seq data were mapped to Shae.V2 genome scaffolds using TopHat2 (v.2.1.0) [60], and gene models were inferred from mapped RNA-Seq data using Cufflinks (v.2.2.1, Cufflinks, RRID:SCR_014597) [61].
(3) Gene models were predicted *ab initio* using AUGUSTUS (v.3.1, Augustus, RRID:SCR_008417) [62], Semi-HMM-based Nucleic Acid Parser (SNAP; v.6.7) [63] and GENEMARK (v.4.2.9, GeneMark, RRID:SCR_011930) [64] with full-length, *de novo*-assembled transcripts (step 1) used for gene model training.
(4) *Ab initio*-gene predictions, gene models inferred from RNA-Seq data, non-redundant transcriptomes, transferred Shae.V1 gene models and genome-aligned predicted proteomes for *S. mansoni* (NCBI BioProject: PRJEA36577) [29] and *S. japonicum* (NCBI BioProject: PRJEA34885) [47] were combined in MAKER2 to create a gene

set.

(5) EVM was used to select reliable gene models by employing modelled gene structures inferred using the *de novo*-assembled transcriptome and PASA2 (v.2.0.2) [65], and by incorporating all gene model evidence inferred by MAKER2.

Concatenated GFF files from MAKER2, EVM, RATT and liftOver were compared to identify overlapping gene models using GFFREAD (v.2.2.1) [66] employing the merge (–m) option. If gene models overlapped with existing Shae.V1 genes, the model with the longest open reading frame (ORF) was defined as being representative and thus retained. Gene models for Shae.V1 that could not be transferred to the Shae.V2 genome using liftOver or RATT were identified by matching them with the most similar gene in the gene sets inferred using MAKER2 and EVM, of which the longest ORF representing the coding region was retained.

Proteins inferred from the merged gene model files that were similar to the Shae.V1 gene set were compared with the predicted proteome of *S. mansoni* using OrthoMCL (v.2.0.4) [67]. *Schistosoma mansoni* proteins that had no predicted ortholog in the transferred Shae.V1 gene set but shared amino acid sequence similarity (BLASTp; v.2.5.1+) [45] with predicted proteins in gene models inferred using MAKER2 and/or EVM were identified. For these gene models, the longest ORF encoding the respective protein sequence was retained in the final gene set.

All retained gene models were merged into a single GFF file. Subsequently, their integrity was confirmed and overlapping gene models were removed using GAG (v.2.0.1) [68] and tbl2asn (v.25.3, tbl2asn, RRID:SCR_016636) [69]. The completeness of the final gene set was assessed by searching for orthologs of 978 conserved gene models representing metazoans, using the program BUSCO in the gene set mode. For comparisons among gene sets, the same analysis was carried out for Shae.V1, *S. mansoni* and *S. japonicum* gene sets (WormBase Parasite version WBPS8).

Determining synteny between genomes

Proteins predicted from the Shae.V1 and Shae.V2 and *S. mansoni* genomes were compared using OrthoMCL, and inferred single-copy orthologs (SCOs) were selected for further processing. The number and order of syntenic blocks containing three or more SCOs was assessed using OrthoCluster [70]. Syntenic scaffolds and comparisons of assembly contiguity and integrity between genomes were displayed as circular plots using Circos (v.0.69-6, Circos, RRID:SCR_011798) [71] and edited using Inkscape (Inkscape, RRID:SCR_014479) [72].

Phylogenetic analysis

Single-copy orthologous groups of genes (n = 410) shared among 14 trematode species (*S. haematobium*, PRJNA78265; *S. bovis*, PRJNA451066 [73]; *S. curassoni*, PRJEB519; *S. mattheei*, PRJEB523; *S. margrebowiei*, PRJEB522; *S. mansoni*, PRJEA36577; *S. rodhaini*, PRJEB526; *S. japonicum*, PRJEA34885; *Trichobilharzia regenti*, PRJEB4662; *Clonorchis sinensis*, PRJNA386618; *Opisthorchis viverrini*, PRJNA222628; *Paragonimus westermani*, PRJNA454344 [74]; *Fasciola hepatica*, PRJNA179522; and *Echinostoma caproni*, PRJEB1207), for which draft genomes were publicly available (*via* NCBI or WormBase ParaSite WBPS13) [75], and a monogenean outgroup (*Gyrodactylus salaris*, PRJNA244375) [76] were identified. The amino acid sequences inferred from these genes were subjected to automated quality improvement for multiple sequence alignment (AQUA; v.1.1) [77]. In brief, alignments were constructed employing the programs MUSCLE (v.3.8.31, MUSCLE, RRID:SCR_011812) [78] and MAFFT (v.7.271, MAFFT, RRID:SCR_011811) [79] and then refined using RASCAL (v.1.34) [80]. Alignments with a score of < 0.8 (NorMD) [81] were optimised and merged into subsets using the program PartitionFinder (v.2.1.1) [82], removing those that did not contain all 20 amino acids and/or those that represented mitochondrial or

viral amino acid replacement matrices. Remaining subsets (*n* = 186) were subjected to analysis using the maximum likelihood (ML) and Bayesian inference (BI) tree-building methods. For ML, analysis of the replacement matrices inferred from each subset in the alignment was conducted using the program RAxML (v.8.2.9, RAxML, RRID:SCR_006086) [83]. For BI, four Markov chains were run for 1,000,000 Markov chain Monte Carlo (MCMC) generations (metropolis-coupled), and trees were sampled every 100 generations using the program MrBayes (v.3.2.6, MrBayes, RRID:SCR_012067) [84], applying the same replacement matrices as used for ML. After discarding the first 25% of trees as burn-in, Bayesian posterior probabilities (BPP) were calculated based on the remaining trees; an analysis was completed when the potential scale reduction factor (PSRF) was ≈ 1 and the average standard deviation of split frequencies was ≈ 0. Trees were displayed using FigTree (v.1.31, FigTree, RRID:SCR_008515) [85].

Improved genome assembly

Approximately three million error-corrected PacBio reads with an average length of 2410 nucleotides (nt) were sequenced from ~ 33 µg of whole genome-amplified DNA, achieving 19-fold coverage of the *S. haematobium* genome (Supplementary Table S1). In addition, 350 million reads (95-fold coverage) were sequenced from the Chicago library (Supplementary Table S1). Following filtering, published Illumina mate-pair and short reads (BioProject: PRJNA78265) [14], corrected PacBio reads and Chicago reads were used to re-scaffold and assemble Shae.V1 contigs into the refined Shae.V2 genome for *S. haematobium* (Table 1). The latter genome was assembled into 666 scaffolds (previously 99,953) with an average length of 556,859 bp (previously 3853 bp), an N50 of 4.8 Mb (previously 0.31 Mb) and an L50 of 26 scaffolds (previously 365). Approximately 23.4% of the genome assembled into scaffolds of > 100,000 bp in length (previously 0.96%), with the longest scaffold containing 14.3 million bp (previously 1.8 million bp) (Table 1 and Fig. 1). In addition, the new assembly was more contiguous, with 15,113 gaps comprised of 950,957 ambiguous nucleotides ('Ns'), representing 0.26% (previously 6.02%) of the genome (Table 1).

A comparison of the Shae.V2 genome to that of Shae.V1 (Fig. 2) or *S. mansoni* (WBPS8) (Fig. 3) inferred 5506 and 218 syntenic regions containing SCOs, respectively. For *S. mansoni*, all eight chromosomes comprising 258,697,509 bp (representing 71% of the entire *S. mansoni* genome) were represented by a total of 79 *S. haematobium* scaffolds comprising 303,401,942 bp (representing 81.7% of the entire *S. haematobium* genome), confirming a high level of completeness of the Shae.V2 assembly (Fig. 3). For Shae.V1, SCOs linked 135 Shae.V2 scaffolds (total length: 361,192,130 bp, representing 97.3% of the Shae.V2 genome; average length: 2,675,500 bp) with 810 Shae.V1 scaffolds (total length: 268,521,193 bp, representing 71.4% of the Shae.V1 genome; average length: 331,508 bp), demonstrating a substantial increase in genome integrity through an ~ 6-fold improvement in the contiguity of the new assembly (Fig. 2).

A comparison of the number of gaps in the portion of the Shae.V2 assembly representing the *S. mansoni* chromosomes (Fig. 3) showed that the improved *S. haematobium* assembly contained less (*n* = 3128) gaps than the *S. mansoni* genome assembly (*n* = 5861), representing a total of 122,623 bp (*S. mansoni*: 1,454,291 bp). Most of the gaps in the Shae.V2 assembly (96.8%) were either 25 bp (*n* = 2636; introduced by PBJelly) or 100 bp (*n* = 391; introduced by HiRise) long, whereas for *S. mansoni*, 92.5% of them were either 200 bp (*n* = 5298) or 2000 bp (*n* = 123) long.

Refined gene set

Gene models from the Shae.V1 gene set were merged and/or refined and successfully transferred to the Shae.V2 genome by consolidating a total of 37,190 inferred gene models.

These models were either predicted by AUGUSTUS (*n* = 2132), EVM (*n* = 9633), GENEMARK (*n* = 161), MAKER2 (*n* = 8310) or SNAP (*n* = 518), or directly inferred from the Shae.V1 gene set by liftOver (*n* = 7244) or RATT (*n* = 9192). The final, merged set included 9314 genes and represented the 11,140 gene models present in the Shae.V1 gene set. In 1081 cases, two or more gene models in Shae.V1 were merged into a single gene model for Shae.V2. In contrast, 76 gene models in Shae.V1 were split into multiple models, representing a total of 178 genes in Shae.V2.

The level of completeness of the Shae.V2 gene set was determined by assessing the presence of 978 BUSCO genes both in the genome (Fig. 4A, B; Table 2) and in the gene set (Fig. 4C, D; Table 2). For both modes of inference (i.e. genome-based and gene set-based) employed in BUSCO, we predicted more complete, single-copy genes and fewer fragmented and missing genes in the Shae.V2 than the Shae.V1 gene set. Comparisons showed that the Shae.V2 gene set was predicted to be nearly as complete as that of *S. mansoni* and substantially more complete than that of *S. japonicum*.

Phylogenetic position of *S. haematobium* in relation to other parasitic trematodes

Phylogenetic analysis of concatenated amino acid sequence data inferred from 186 SCOs using BI and ML tree-building methods confirmed the phylogenetic position of *S. haematobium* relative to other representatives of the class Trematoda for which draft genomes were available in public databases. Clades representing the orders Plagiorchiida (intestinal fluke *E. caproni*, liver fluke *F. hepatica*, and lung fluke *P. westermani*) and Opistorchiida (liver flukes *C. sinensis* and *O. viverrini*) were basal to the family Schistosomatidae (blood flukes) [73, 74]. Within the schistosome clade, *T. regenti* (bird schistosome) was located basal to the genus *Schistosoma*, which was divided into the Asian clade (represented by *S. japonicum*), the *S. mansoni* group (represented by *S. mansoni* and *S. rodhaini*) and the *S. haematobium* group [86]. The five representatives of the latter group included here were very closely interrelated, consistent with previous phylogenetic analyses and with the ability of some species to cross-hybridise [73, 86].

Discussion

Short-read sequencing technologies have enabled the sequencing of genomes for a plethora of organisms, including those of complex eukaryotic pathogens, to a high-quality draft status [14, 29, 42]. Although useful, most draft genomes are fragmented, and substantial efforts are now required to achieve more contiguous assemblies. Recently, long-read technologies have substantially improved our prospects to define accurate genomes for eukaryotic organisms [24, 42, 87, 88]. Here, we harnessed long-read and long-range sequencing, together with existing short-read data, to achieve a substantially enhanced genome assembly for *S. haematobium* that is comparable or even superior to those for related schistosome species (Figs 1 and 3). As the quality of a genome assembly has a substantial impact on downstream analyses, in particular gene annotation and SNP-calling [89, 90], this improved genomic resource will accelerate systems biological research of *S. haematobium* and related schistosomes.

By combining established gene (re-)annotation pipelines [25, 33] and by incorporating evidence from closely related species for which high-quality genomes and gene sets were available, we inferred a gene set that is as complete as that of *S. mansoni*, based on the analysis of conserved SCOs. Importantly, by employing a gene transfer approach, instead of re-predicting the complete gene set *de novo*, we retained gene models curated previously for *S. haematobium*, including those coding for key families of proteins, such as kinases [19] and GPCRs [18]. In addition, a synteny analysis employing the improved gene set revealed that,

overall, there is concordance between the improved assembly for *S. haematobium* and that of *S. mansoni*.

Despite this concordance, we identified some differences. For example, the Shae.V2 gene set is ~ 8% smaller than that of *S. mansoni*, and ~ 16% smaller than Shae.V1. The higher number of gene models in Shae.V1 might be explained by a more fragmented assembly, resulting in the prediction of more, incomplete gene models. This proposal is supported by significantly shorter genes (mean: 11,907 bp; median: 5773 bp) for Shae.V1 compared with Shae.V2 (mean: 18,332 bp; median: 11,759), and by the finding that genes predicted at the start or end of a scaffold were, on average, significantly shorter for Shae.V1 than for Shae.V2 (Fig. 6). The lower number of fragmented BUSCO genes identified in Shae.V2 compared with Shae.V1 lends additional support to this hypothesis. Our findings here are consistent with results for *S. mansoni*, where a substantial improvement [29] of the initial draft genome [91] led to hundreds of merged or discarded gene models and, overall, to a reduced number of predicted genes.

For the most recent *S. mansoni* gene set (WBPS11), both the average length of genes (21,785 bp) and number of genes (n = 10,131) are higher than for Shae.V2, suggesting a more complete assembly and gene set. However, the length distribution of genes is comparable between the two species, and contrasts that for Shae.V1, which shows a clear bias toward shorter genes (Fig. 6). Furthermore, it is plausible that the size of the gene set and the average gene length for *S. mansoni* are higher than for Shae.V2, because additional RNA-Seq data available for *S. mansoni* (e.g., for the cercarial stage) provided evidence for minimally or selectively expressed transcripts, thus facilitating the detection of novel gene models [26, 29]. In the future, additional RNA-Seq data from multiple developmental stages (including miracidia, sporocysts and cercariae), for which data are currently unavailable, as well as long-read RNA-Seq data (cf. [92]), should assist in the curation of gene models and the discovery of new transcripts for *S. haematobium*. Another possible reason for a smaller inferred gene set might relate to the gene transfer approach employed here [51, 52] that did not include *de novo* prediction of genes in regions that previously did not have gene annotations.

In addition to the observed differences between the two most complete schistosome gene sets (*S. mansoni* and now *S. haematobium*), we also detected a number of differences in the associated genome assemblies (Fig. 3). For instance, *S. haematobium* scaffolds that contained gaps (e.g., scaffolds 1, 134, 153 and 257) tended to align to multiple (n = 2-6) distinct *S. mansoni* chromosomes, suggesting mis-assemblies. Similarly, there were scaffolds without gaps in the *S. haematobium* assembly (e.g., scaffolds 109, 142 and 149) which corresponded to multiple regions in distinct *S. mansoni* chromosomes that contained gaps, suggesting some incorrect scaffolding in the *S. mansoni* assembly. However, in both cases, it is possible that such regions do differ between the two species and are indeed the result of genome rearrangements. Whether these discrepancies represent mis-assemblies or stem from genomic rearrangement events could be the subject of comparative investigations using additional long-read sequencing in the future.

The goal here was to provide a high-quality genomic resource for *S. haematobium*, which will enable in-depth gene (re-)annotation employing short- and long-read RNA-Seq data and, more broadly, serve as a reference for functional and population genomics investigations of schistosomes. Overall, despite some differences in gene numbers and scaffold synteny, the BUSCO analysis presented here demonstrated and confirmed a step-change improvement in contiguity for the *S. haematobium* genome assembly and for the gene set, compared with the first draft (Shae.V1). Also, it provided evidence for an assembly quality that is comparable to the best available genome for *S. mansoni* [29]. Achieving a chromosome-contiguous assembly is the ultimate goal, which will provide substantial benefits to the research community, and

should underpin systems biological investigations and the discovery of new disease interventions.

Availability of supporting data

The genome assembly and gene set are available from NCBI (BioProject: PRJNA78265) and all associated raw read data are available from the Sequence Read Archive (SRA) under the accession numbers SRR8485134-SRR8485168. All supporting data and materials are available in the *GigaScience* GigaDB database [93].

Additional file

Supplementary Table S1.xlsx

Abbreviations

bp: base pair; BI: Bayesian inference; BPP: Bayesian posterior probabilities; BUSCO: Benchmarking Universal Single-Copy Orthologs; GFF: General Feature Format; GPCRs: G protein-coupled receptors; kb: kilobase pair; Mb: megabase pair; MCMC: Markov chain Monte Carlo; ML: maximum likelihood; NCBI: National Center for Biotechnology Information; ORF: open reading frame; PacBio: Pacific Biosciences; PSRF: potential scale reduction factor; RNA-Seq: RNA sequencing; SCOs: single-copy orthologs; SNP: single-nucleotide polymorphism; WGA: whole-genome amplification.

Ethics approval and consent to participate

Not applicable

Consent for publication

Not applicable

Competing interests

The authors declare that they have no competing interests.

Funding

Support from the National Health and Medical Research Council (NHMRC) of Australia, the Australian Research Council and Melbourne Water Corporation, The University of Melbourne (BIP) (R.B.G.) and the National Cancer Institute, National Institutes of Health, USA (award R01CA164719) (P.J.B) is gratefully acknowledged. P.K.K. holds an NHMRC Early Career Research Fellowship. N.D.Y. holds an NHMRC Career Development Fellowship.

Authors' contributions

B.W, D.R, P.J.B, R.B.G and N.D.Y. designed the study and acquired funding. B.W, D.R and P.J.B provided material for sequencing through the NIAID Schistosomiasis Resource Center, at the Biomedical Research Institute, Rockville, Maryland, for distribution through BEI Resources, NIH-NIAID Contract HHSN272201000005I. T.M.C, Y.L.L and K.G.C carried out PacBio sequencing. A.J.S, P.K.K., N.D.Y carried out genome assembly, gene prediction and all other analyses. A.J.S, R.B.G and N.D.Y wrote the manuscript with contributions from all co-authors.

Acknowledgements

We thank Vaughan R. Southgate (Natural History Museum, London, United Kingdom) for providing the schistosome image.

References

1. Steinmann P, Keiser J, Bos R, et al. Schistosomiasis and water resources development: systematic review, meta-analysis, and estimates of people at risk. Lancet Infect Dis. 2006;**6**:411-25.
2. Gryseels B, Polman K, Clerinx J, et al. Human schistosomiasis. Lancet. 2006;**368**:1106-18.
3. Smith JH, Christie JD. The pathobiology of *Schistosoma haematobium* infection in humans. Hum Pathol. 1986;**17**:333-45.
4. Andrade ZA. Schistosomiasis and liver fibrosis. Parasite Immunol. 2009;**31**:656-63.
5. Jourdan PM, Holmen SD, Gundersen SG, et al. HIV target cells in *Schistosoma haematobium*-infected female genital mucosa. Am J Trop Med Hyg. 2011;**85**:1060-4.
6. Botelho MC, Alves H, Barros A, et al. The role of estrogens and estrogen receptor signaling pathways in cancer and infertility: the case of schistosomes. Trends Parasitol. 2015;**31**:246-50.
7. Palumbo E. Association between schistosomiasis and cancer: a review. Infect Dis Clin Pract. 2007;**15**:145-8.
8. Tebeje BM, Harvie M, You H, et al. Schistosomiasis vaccines: where do we stand? Parasit Vectors. 2016;**9**:528.
9. Doenhoff MJ, Hagan P, Cioli D, et al. Praziquantel: its use in control of schistosomiasis in sub-Saharan Africa and current research needs. Parasitology. 2009;**136**:1825-35.
10. Rollinson D, Knopp S, Levitz S, et al. Time to set the agenda for schistosomiasis elimination. Acta Trop. 2013;**128**:423-40.
11. Rollinson D. A wake up call for urinary schistosomiasis: reconciling research effort with public health importance. Parasitology. 2009;**136**:1593-610.
12. World-Health-Organization. Sustaining the drive to overcome the global impact of neglected tropical diseases. Second WHO Report on Neglected Tropical Diseases. Geneva, Switzerland. 2013.
13. Lewis FA, Liang YS, Raghavan N, et al. The NIH-NIAID schistosomiasis resource center. PLoS Negl Trop Dis. 2008;**2**:e267.
14. Young ND, Jex AR, Li B, et al. Whole-genome sequence of *Schistosoma haematobium*. Nat Genet. 2012;**44**:221-5.
15. Cantacessi C, Hofmann A, Young ND, et al. Insights into SCP/TAPS proteins of liver flukes based on large-scale bioinformatic analyses of sequence datasets. PLoS One. 2012;**7**:e31164.
16. Cantacessi C, Seddon JM, Miller TL, et al. A genome-wide analysis of annexins from parasitic organisms and their vectors. Sci Rep. 2013;**3**:2893.
17. Zerlotini A, Aguiar ER, Yu F, et al. SchistoDB: an updated genome resource for the three key schistosomes of humans. Nucleic Acids Res. 2013;**41**:D728-31.
18. Campos TD, Young ND, Korhonen PK, et al. Identification of G protein-coupled receptors in *Schistosoma haematobium* and *S. mansoni* by comparative genomics. Parasit Vectors. 2014;**7**:242.
19. Stroehlein AJ, Young ND, Jex AR, et al. Defining the *Schistosoma haematobium* kinome enables the prediction of essential kinases as anti-schistosome drug targets. Sci Rep. 2015;**5**:17759.
20. Fu CL, Odegaard JI, De'Broski RH, et al. A novel mouse model of *Schistosoma haematobium* egg-induced immunopathology. PLoS Pathog. 2012;**8**:e1002605.
21. Rinaldi G, Okatcha TI, Popratiloff A, et al. Genetic manipulation of *Schistosoma haematobium,* the neglected schistosome. PLoS Negl Trop Dis. 2011;**5**:e1348.

22. Botelho MC, Vale N, Gouveia MJ, et al. Tumour-like phenotypes in urothelial cells after exposure to antigens from eggs of *Schistosoma haematobium*: an oestrogen-DNA adducts mediated pathway? Int J Parasitol. 2013;**43**:17-26.
23. Rinaldi G, Young ND, Honeycutt JD, et al. New research tools for urogenital schistosomiasis. J Infect Dis. 2015;**211**:861-9.
24. Roberts RJ, Carneiro MO, Schatz MC. The advantages of SMRT sequencing. Genome Biol. 2013;**14**:405.
25. Korhonen PK, Young ND, Gasser RB. Making sense of genomes of parasitic worms: tackling bioinformatic challenges. Biotechnol Adv. 2016;**34**:663-86.
26. Mudge JM, Harrow J. The state of play in higher eukaryote gene annotation. Nat Rev Genet. 2016;**17**:758-72.
27. DeMarco R, Mathieson W, Manuel SJ, et al. Protein variation in blood-dwelling schistosome worms generated by differential splicing of micro-exon gene transcripts. Genome Res. 2010;**20**:1112-21.
28. Yandell M, Ence D. A beginner's guide to eukaryotic genome annotation. Nat Rev Genet. 2012;**13**:329-42.
29. Protasio AV, Tsai IJ, Babbage A, et al. A systematically improved high quality genome and transcriptome of the human blood fluke *Schistosoma mansoni*. PLoS Negl Trop Dis. 2012;**6**:e1455.
30. Tsai IJ, Zarowiecki M, Holroyd N, et al. The genomes of four tapeworm species reveal adaptations to parasitism. Nature. 2013;**496**:57-63.
31. Huang Y, Chen W, Wang X, et al. The carcinogenic liver fluke, *Clonorchis sinensis*: new assembly, reannotation and analysis of the genome and characterization of tissue transcriptomes. PLoS One. 2013;**8**:e54732.
32. Eid J, Fehr A, Gray J, et al. Real-time DNA sequencing from single polymerase molecules. Science. 2009;**323**:133-8.
33. Stroehlein AJ, Young ND, Gasser RB. Improved strategy for the curation and classification of kinases, with broad applicability to other eukaryotic protein groups. Sci Rep. 2018;**8**:6808.
34. Putnam NH, O'Connell BL, Stites JC, et al. Chromosome-scale shotgun assembly using an *in vitro* method for long-range linkage. Genome Res. 2016;**26**:342-50.
35. BBMap short read aligner and other bioinformatic tools. 2019. http://sourceforge.net/projects/bbmap/. Accessed 17 Apr 2019.
36. Salmela L, Rivals E. LoRDEC: accurate and efficient long read error correction. Bioinformatics. 2014;**30**:3506-14.
37. Bolger AM, Lohse M, Usadel B. Trimmomatic: a flexible trimmer for Illumina sequence data. Bioinformatics. 2014;**30**:2114-20.
38. Zaharia M, Bolosky WJ, Curtis K, et al. Faster and more accurate sequence alignment with SNAP. arXiv. 2011. https://arxiv.org/abs/1111.5572.
39. Li H, Handsaker B, Wysoker A, et al. The sequence alignment/map format and SAMtools. Bioinformatics. 2009;**25**:2078-9.
40. Picard tools. 2019. http://broadinstitute.github.io/picard. Accessed 17 Apr 2019.
41. Huang S, Chen Z, Huang G, et al. HaploMerger: reconstructing allelic relationships for polymorphic diploid genome assemblies. Genome Res. 2012;**22**:1581-8.
42. English AC, Richards S, Han Y, et al. Mind the gap: upgrading genomes with Pacific Biosciences RS long-read sequencing technology. PLoS One. 2012;**7**:e47768.
43. Boetzer M, Pirovano W. SSPACE-LongRead: scaffolding bacterial draft genomes using long read sequence information. BMC Bioinformatics. 2014;**15**:211.
44. NCBI Nucleotide. 2019. https://www.ncbi.nlm.nih.gov/nucleotide. Accessed 17 Apr 2019.

45. Camacho C, Coulouris G, Avagyan V, et al. BLAST+: architecture and applications. BMC Bioinformatics. 2009;**10**:421.
46. Waterhouse RM, Seppey M, Simao FA, et al. BUSCO applications from quality assessments to gene prediction and phylogenomics. Mol Biol Evol. 2017;**35**:543–8.
47. *Schistosoma japonicum* Genome Sequencing and Functional Analysis Consortium. The *Schistosoma japonicum* genome reveals features of host-parasite interplay. Nature. 2009;**460**:345-51.
48. Shen W, Le S, Li Y, et al. SeqKit: a cross-platform and ultrafast toolkit for FASTA/Q file manipulation. PLoS One. 2016;**11**:e0163962.
49. Chaisson MJ, Tesler G. Mapping single molecule sequencing reads using basic local alignment with successive refinement (BLASR): application and theory. BMC Bioinformatics. 2012;**13**:238.
50. Ramirez F, Ryan DP, Gruning B, et al. deepTools2: a next generation web server for deep-sequencing data analysis. Nucleic Acids Res. 2016;**44**:W160-5.
51. Kuhn RM, Haussler D, Kent WJ. The UCSC genome browser and associated tools. Brief Bioinform. 2013;**14**:144-61.
52. Otto TD, Dillon GP, Degrave WS, et al. RATT: Rapid Annotation Transfer Tool. Nucleic Acids Res. 2011;**39**:e57.
53. Tarailo-Graovac M, Chen N. Using RepeatMasker to identify repetitive elements in genomic sequences. Curr Protoc Bioinformatics. 2009;**25**:4.10.1-4.10.14.
54. Harris RS: Improved pairwise alignment of genomic DNA. 2007. https://www.bx.psu.edu/~rsharris/lastz/. Accessed 17 Apr 2019.
55. Package recipe 'ucsc-chainnet'. 2019. https://bioconda.github.io/recipes/ucsc-chainnet/README.html. Accessed 17 Apr 2019.
56. Holt C, Yandell M. MAKER2: an annotation pipeline and genome-database management tool for second-generation genome projects. BMC Bioinformatics. 2011;**12**:491.
57. Haas BJ, Salzberg SL, Zhu W, et al. Automated eukaryotic gene structure annotation using EVidenceModeler and the Program to Assemble Spliced Alignments. Genome Biol. 2008;**9**:R7.
58. Haas BJ, Papanicolaou A, Yassour M, et al. *De novo* transcript sequence reconstruction from RNA-Seq using the Trinity platform for reference generation and analysis. Nat Protoc. 2013;**8**:1494-512.
59. TransDecoder. 2019. https://github.com/TransDecoder/TransDecoder/wiki. Accessed 17 Apr 2019.
60. Trapnell C, Pachter L, Salzberg SL. TopHat: discovering splice junctions with RNA-Seq. Bioinformatics. 2009;**25**:1105-11.
61. Trapnell C, Roberts A, Goff L, et al. Differential gene and transcript expression analysis of RNA-seq experiments with TopHat and Cufflinks. Nat Protoc. 2012;**7**:562-78.
62. Stanke M, Morgenstern B. AUGUSTUS: a web server for gene prediction in eukaryotes that allows user-defined constraints. Nucleic Acids Res. 2005;**33**:W465-7.
63. Korf I. Gene finding in novel genomes. BMC Bioinformatics. 2004;**5**:59.
64. Borodovsky M, Lomsadze A, Ivanov N, et al. Eukaryotic gene prediction using GeneMark.hmm. Curr Protoc Bioinformatics. 2003;**35**:4.6.1-4.6.10.
65. Haas BJ, Delcher AL, Mount SM, et al. Improving the *Arabidopsis* genome annotation using maximal transcript alignment assemblies. Nucleic Acids Res. 2003;**31**:5654-66.
66. Pertea G: 2019. https://github.com/gpertea/gffread. Accessed 17 Apr 2019.
67. Li L, Stoeckert Jr. CJ, Roos DS. OrthoMCL: identification of ortholog groups for eukaryotic genomes. Genome Res. 2003;**13**:2178-89.

68. Geib SM, Hall B, Derego T, et al. Genome Annotation Generator: a simple tool for generating and correcting WGS annotation tables for NCBI submission. GigaScience. 2018;**7**:1-5.
69. Kans JA, Ouellette BF. Submitting DNA sequences to the databases. Meth Biochem Anal. 2001;**43**:65-81.
70. Vergara IA, Chen N. Using OrthoCluster for the detection of synteny blocks among multiple genomes. Curr Protoc Bioinformatics. 2009;**27**:6.10.1-6.10.18.
71. Krzywinski M, Schein J, Birol I, et al. Circos: an information aesthetic for comparative genomics. Genome Res. 2009;**19**:1639-45.
72. Inkscape. 2019. https://inkscape.org. Accessed 17 Apr 2019.
73. Oey H, Zakrzewski M, Gravermann K, et al. Whole-genome sequence of the bovine blood fluke *Schistosoma bovis* supports interspecific hybridization with *S. haematobium*. PLoS Pathog. 2019;**15**:e1007513.
74. Oey H, Zakrzewski M, Narain K, Devi KR, Agatsuma T, Nawaratna S, Gobert GN, Jones MK, Ragan MA, McManus DP, Krause L. Whole-genome sequence of the oriental lung fluke *Paragonimus westermani*. Gigascience. 2019 Jan 1;8(1). doi: 10.1093/gigascience/giy146.
75. Lee RYN, Howe KL, Harris TW, et al. WormBase 2017: molting into a new stage. Nucleic Acids Res. 2018;**46**:D869-74.
76. Hahn C, Fromm B, Bachmann L. Comparative genomics of flatworms (platyhelminthes) reveals shared genomic features of ecto- and endoparastic neodermata. Genome Biol Evol. 2014;**6**:1105-17.
77. Muller J, Creevey CJ, Thompson JD, et al. AQUA: automated quality improvement for multiple sequence alignments. Bioinformatics. 2010;**26**:263-5.
78. Edgar RC. MUSCLE: multiple sequence alignment with high accuracy and high throughput. Nucleic Acids Res. 2004;**32**:1792-7.
79. Katoh K, Standley DM. MAFFT multiple sequence alignment software version 7: improvements in performance and usability. Mol Biol Evol. 2013;**30**:772-80.
80. Thompson JD, Thierry JC, Poch O. RASCAL: rapid scanning and correction of multiple sequence alignments. Bioinformatics. 2003;**19**:1155-61.
81. Thompson JD, Plewniak F, Ripp R, et al. Towards a reliable objective function for multiple sequence alignments. J Mol Biol. 2001;**314**:937-51.
82. Lanfear R, Frandsen PB, Wright AM, et al. PartitionFinder 2: new methods for selecting partitioned models of evolution for molecular and morphological phylogenetic analyses. Mol Biol Evol. 2017;**34**:772-3.
83. Stamatakis A, Ludwig T, Meier H. RAxML-III: a fast program for maximum likelihood-based inference of large phylogenetic trees. Bioinformatics. 2005;**21**:456-63.
84. Ronquist F, Teslenko M, van der Mark P, et al. MrBayes 3.2: efficient Bayesian phylogenetic inference and model choice across a large model space. Syst Biol. 2012;**61**:539-42.
85. FigTree. 2019. http://tree.bio.ed.ac.uk/software/figtree/. Accessed 17 Apr 2019.
86. Webster BL, Southgate VR, Littlewood DT. A revision of the interrelationships of *Schistosoma* including the recently described *Schistosoma guineensis*. Int J Parasitol. 2006;**36**:947-55.
87. Reuter JA, Spacek DV, Snyder MP. High-throughput sequencing technologies. Mol Cell. 2015;**58**:586-97.
88. Kingan SB, Heaton H, Cudini J, et al. A high-quality *de novo* genome assembly from a single mosquito using PacBio sequencing. Genes (Basel). 2019;**10**:62.

89. Florea L, Souvorov A, Kalbfleisch TS, et al. Genome assembly has a major impact on gene content: a comparison of annotation in two *Bos taurus* assemblies. PLoS One. 2011;**6**:e21400.
90. Watson M, Warr A. Errors in long-read assemblies can critically affect protein prediction. Nat Biotechnol. 2019;**37**:124-6.
91. Berriman M, Haas BJ, LoVerde PT, et al. The genome of the blood fluke *Schistosoma mansoni*. Nature. 2009;**460**:352-8.
92. Magrini V, Gao X, Rosa BA, et al. Improving eukaryotic genome annotation using single molecule mRNA sequencing. BMC Genomics. 2018;**19**:172.
93. Stroehlein AJ; Korhonen PK; Chong TM; Lim YL; Chan KG; Webster B; Rollinson D; Brindley PJ; Gasser RB; Young ND (2019): Supporting data for "High-quality Schistosoma haematobium genome achieved by single-molecule and long-range sequencing" GigaScience Database. http://dx.doi.org/10.5524/100634.

Table 1. Characteristics of the version 2 (Shae.V2) and version 1 (Shae.V1) *Schistosoma haematobium* genomes.

	**Shae.V2**	**Shae.V1**
Number of scaffolds	666	99,953
Total length of all scaffolds	371,394,055	385,110,549
Range of scaffold lengths	518 - 14,276,808	100 - 1,826,302
Mean scaffold length	557,649	3853
Median scaffold length	5586	142
Percentage of scaffolds > 100 kb*	23.42	0.96
Percentage of scaffolds > 1 Mb	13.66	0.02
Percentage of scaffolds > 10 Mb	0.75	0
Scaffold N50	4,779,868	306,738
Scaffold L50	26	365
GC content (excluding Ns)	34.53%	32.19%
Ambiguous bp (Ns)	0.26%	6.02%

* nucleotides

Table 2. Assessment of schistosome genome and gene set completeness through the identification of 978 curated, single-copy, metazoan genes (BUSCOs).

	Complete BUSCOs (C)	Complete and single-copy BUSCOs (S)	Complete and duplicated BUSCOs (D)	Fragmented BUSCOs (F)	Missing BUSCOs (M)
Genome					
Schistosoma haematobium version 2	716 (73.22%)	696 (71.17%)	20 (2.05%)	59 (6.04%)	203 (20.76%)
Schistosoma haematobium version 1	704 (71.99%)	683 (69.84%)	21 (2.15%)	60 (6.14%)	214 (21.89%)
Schistosoma mansoni	704 (71.99%)	691 (70.66%)	13 (1.33%)	58 (5.94%)	216 (22.09%)
Schistosoma japonicum	606 (61.97%)	594 (60.74%)	12 (1.23%)	101 (10.33%)	271 (27.71%)
Gene set					
Schistosoma haematobium version 2	749 (76.59%)	734 (75.06%)	15 (1.54%)	65 (6.65%)	164 (16.77%)
Schistosoma haematobium version 1	708 (72.4%)	691 (70.66%)	17 (1.74%)	115 (11.76%)	155 (15.85%)
Schistosoma mansoni	773 (79.04%)	754 (77.1%)	19 (1.95%)	74 (7.57%)	131 (13.4%)
Schistosoma japonicum	661 (67.59%)	644 (65.85%)	17 (1.74%)	146 (14.93%)	171 (17.49%)

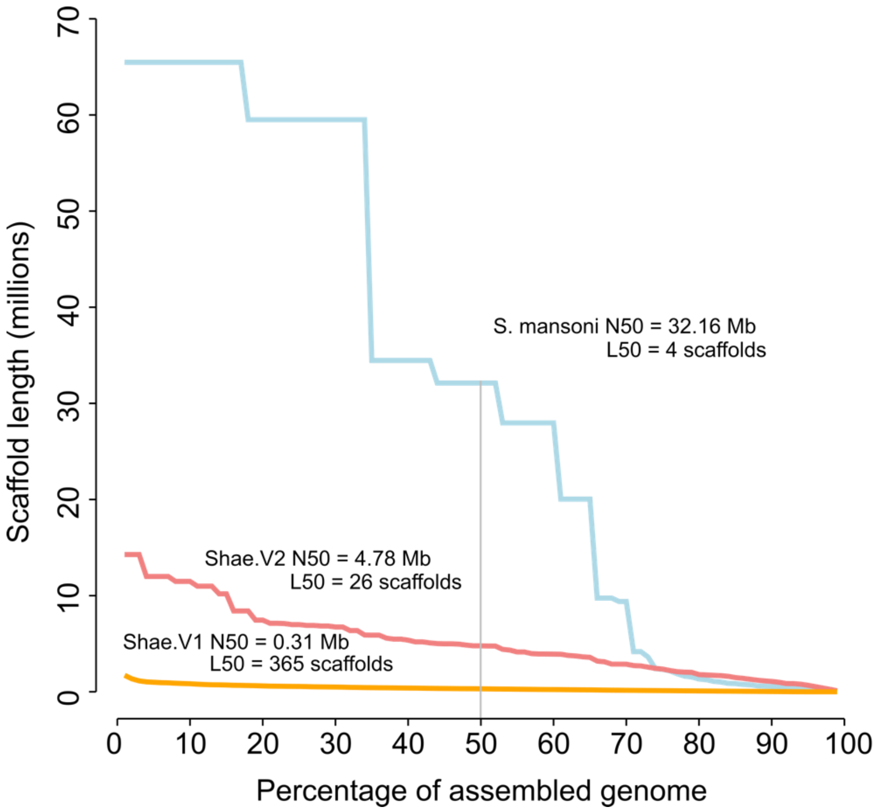

Figure 1: Comparison of schistosome genome assembly quality metrics. Scaffold lengths, N50 and L50 values for *Schistosoma haematobium* genome version 2 (Shae.V2), version 1 (Shae.V1) and *S. mansoni* are shown.

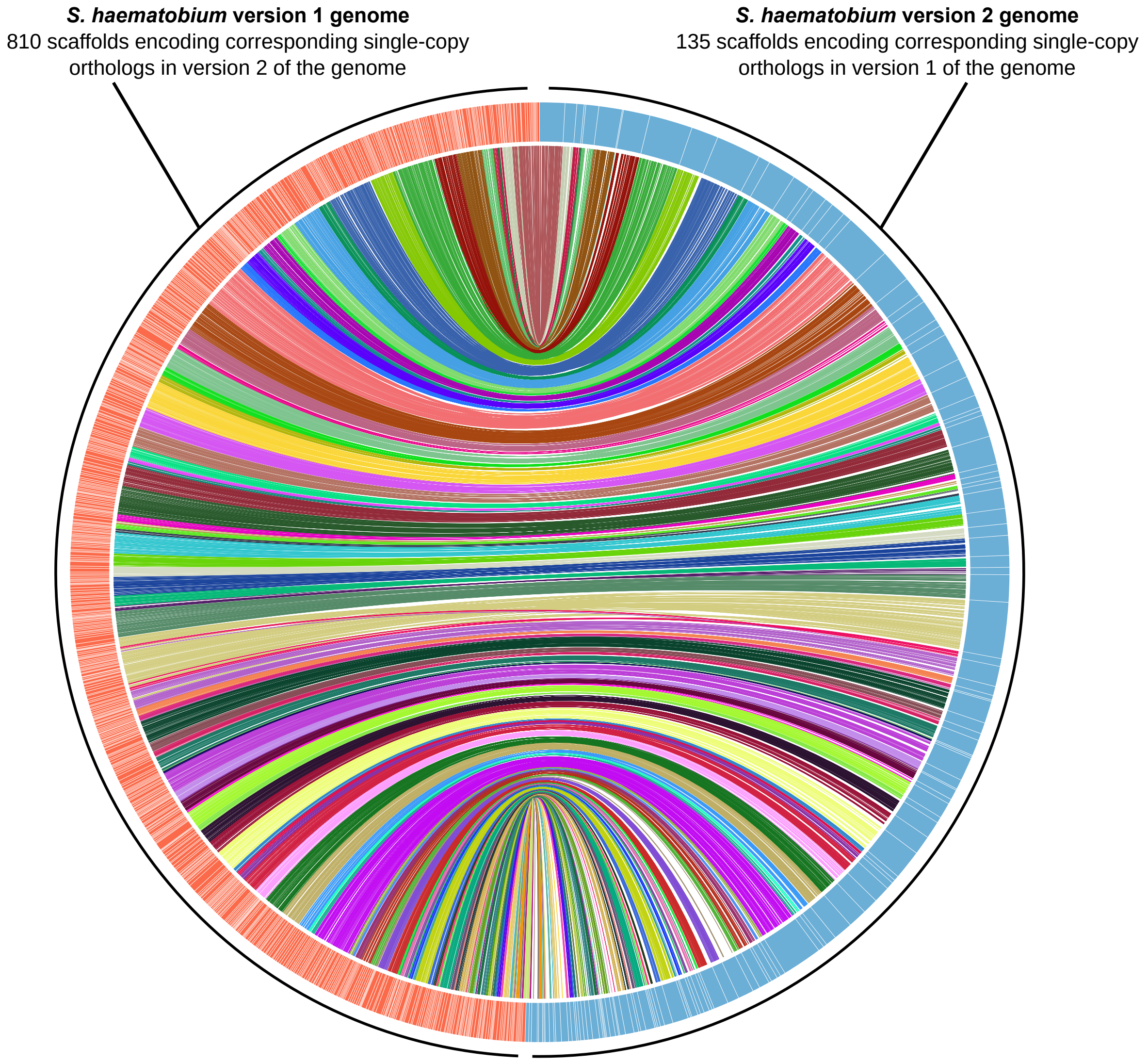

Figure 2: Comparison of the synteny and contiguity of assemblies for *S. haematobium* version 1 (Shae.V1) and version 2 (Shae.V2) genomes. Shae.V1 scaffolds (*n* = 810) are represented by orange bars and are linked with 135 Shae.V2 scaffolds (light blue bars). Scaffolds are arranged as a circular plot based on 5506 regions containing single-copy orthologs (SCOs, each represented by a line connection an orange with a blue scaffold). SCO lines have distinct colours for each Shae.V2 scaffold.

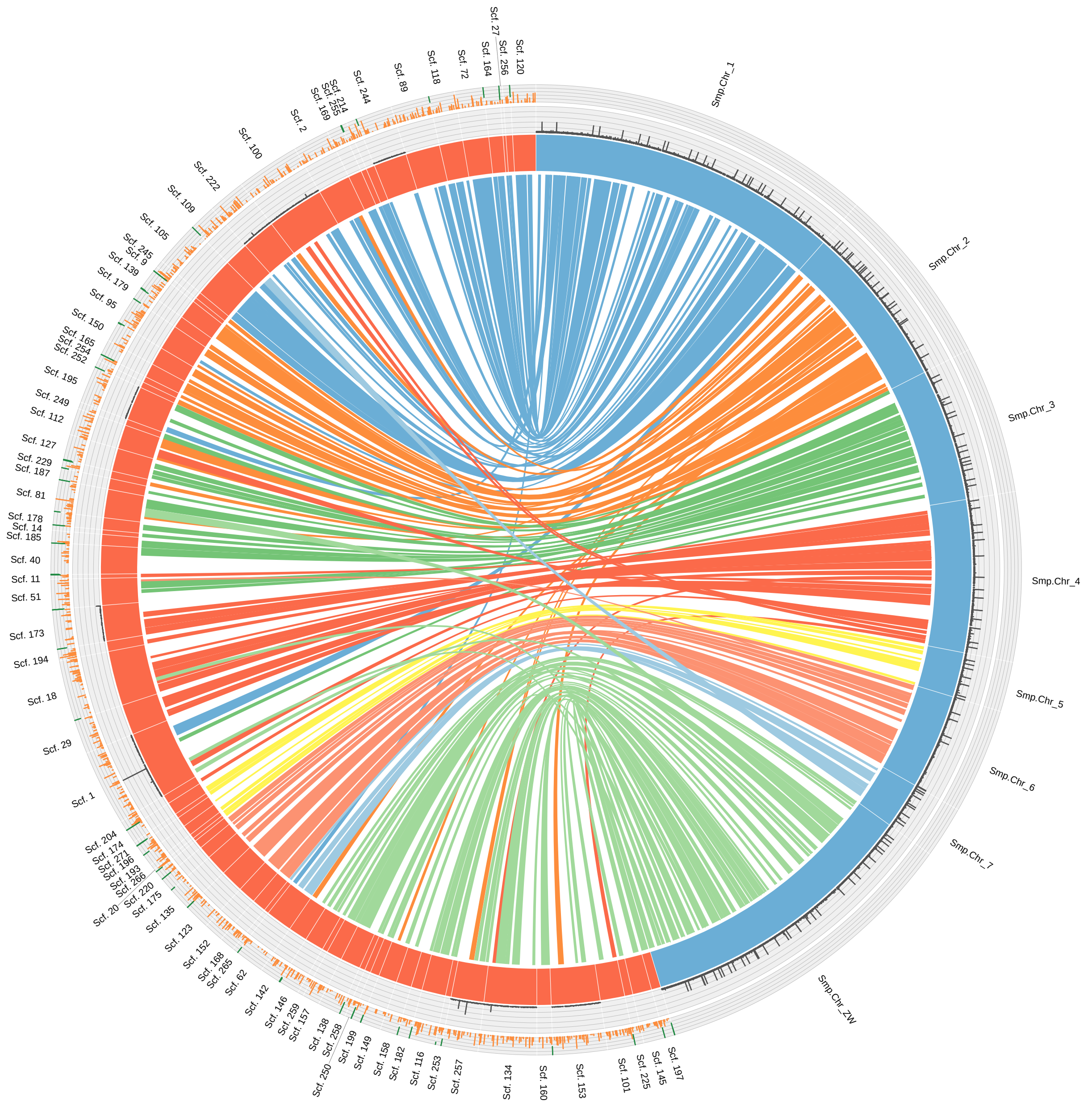

Figure 3: Comparison of the synteny, contiguity and integrity of assemblies for *S. haematobium* version 2 (Shae.V2) and *S. mansoni* (WBPS8). Shae.V2 scaffolds (n = 79) are represented by orange bars and are linked with eight *S. mansoni* chromosomes (light blue bars). Scaffolds are arranged in a circular plot based on 218 regions containing single-copy orthologs (SCOs, each represented by a line connection an orange with a blue scaffold). SCO lines have distinct colours for each *S. mansoni* chromosome. Additionally, gaps ('Ns') are represented as black histograms on a separate track, with the Y-axis representing the size of the region containing ambiguous nucleotides (range 0-5013). On the outer track, orange histograms represent areas of > 1000 bp in length for which the coverage of 'properly paired' reads was less than five reads. Higher histograms represent longer regions. Dark green histograms within the same track represent regions of low 'physical' coverage. The lower the histograms 'drop' from the top of the track, the larger is the size of the regions that have 'physical' coverage of less than ten reads.

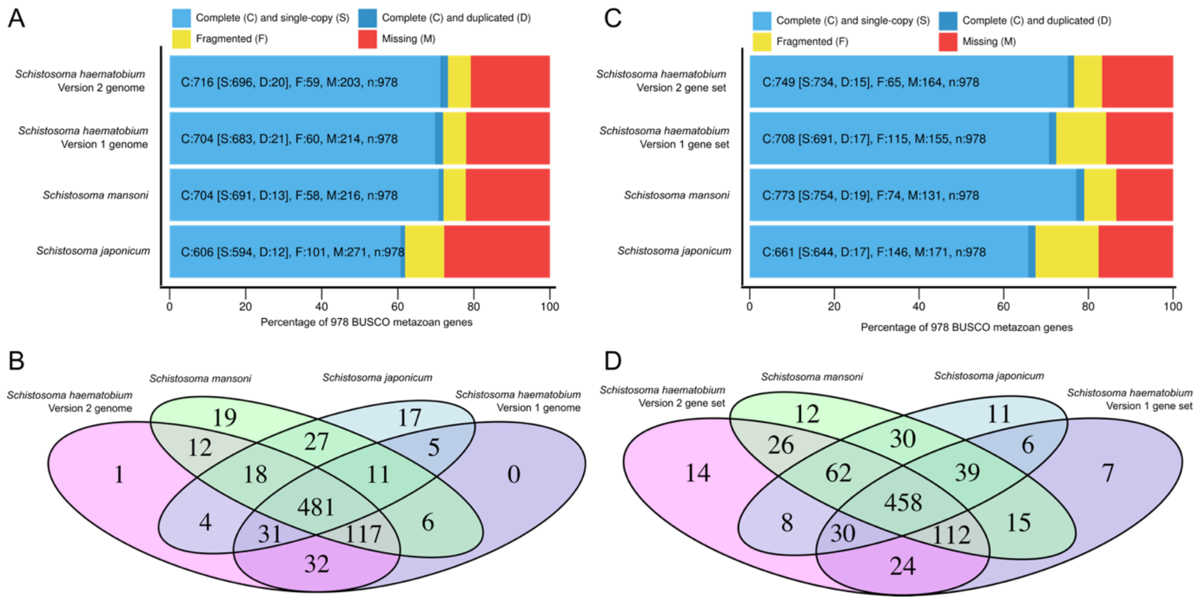

Figure 4: Assessment of genome completeness based on the identification of 978 curated, single-copy, metazoan genes in genomes (A, B) and gene sets (C, D) for schistosomes, using the program BUSCO. The proportion of BUSCO genes identified as complete (single or duplicated), fragmented or missing (genome mode: A; gene set mode: C) and the number of predicted gene models homologous to complete BUSCO genes (genome mode: B; gene set mode: D) are shown for each genome.

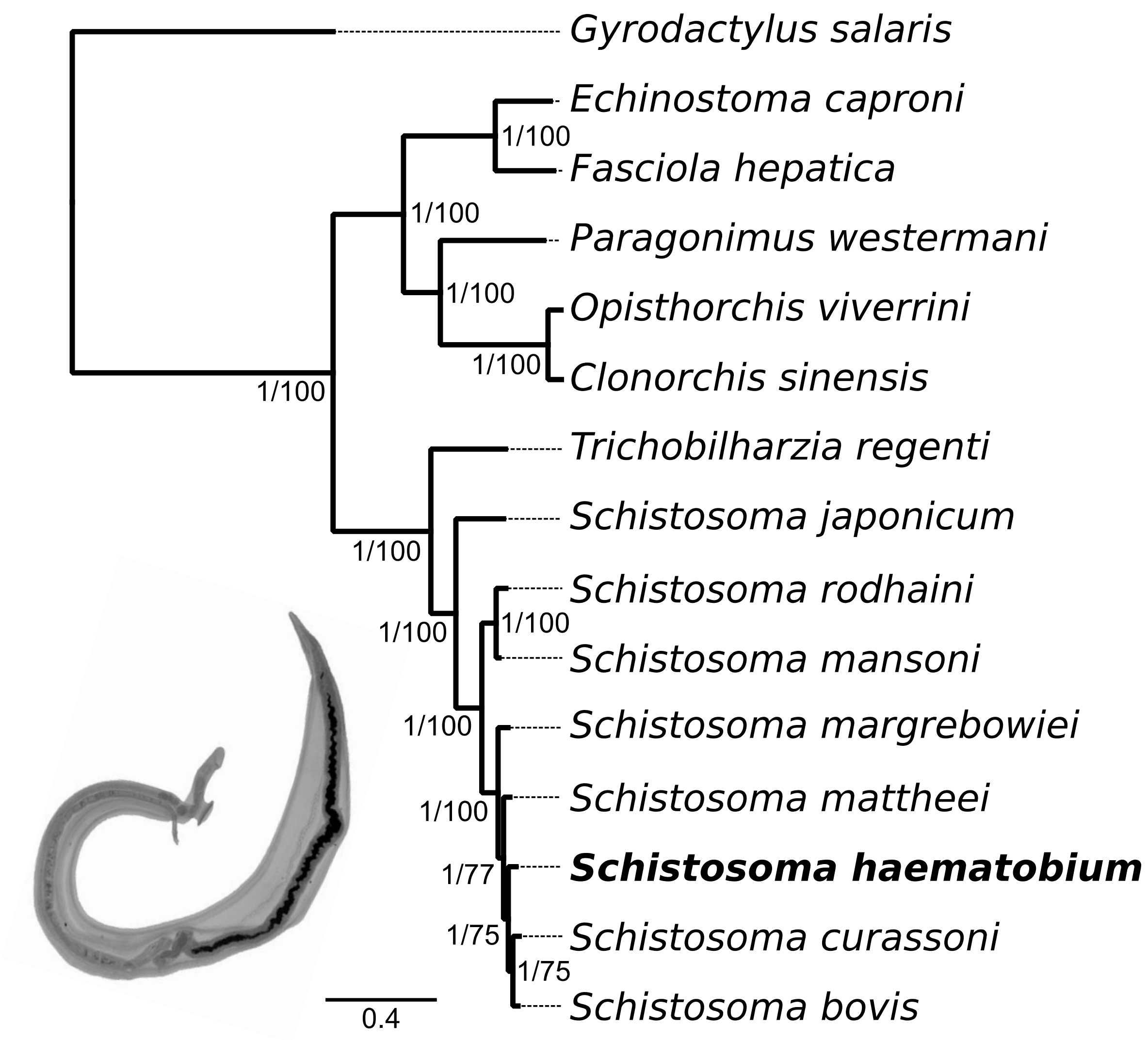

Figure 5: Phylogenetic position of *Schistosoma haematobium* relative to other representatives of the class Trematoda, for which draft genomes were available. Trees constructed using Bayesian inference (BI, shown) and maximum likelihood (ML) analyses of amino acid sequence data inferred from 186 single-copy orthologs (SCOs) had the same topology. Nodal support values for BI and ML analyses are indicated at each branch (posterior probability/bootstrap support). Branch lengths represent the numbers of amino acid substitutions per site at aligned positions. *Gyrodactylus salaris* (class Monogenea) represents the outgroup. Inset image shows a pair of adult schistosomes.

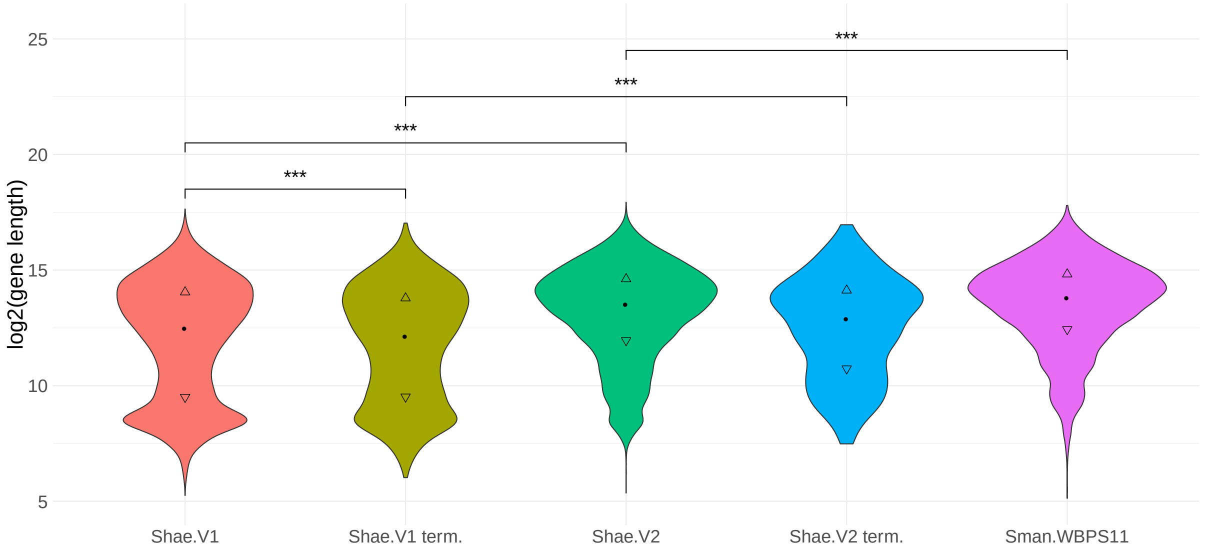

Figure 6: Distribution of gene length for gene sets representing *Schistosoma haematobium* (Shae.V1 and Shae.V2) and *S. mansoni* (Sman.WBPS11). Additionally, distributions are shown for terminal genes (i.e. genes encoded at the start or end of a scaffold) for both *S. haematobium* gene sets ("Shae.V1 term." and "Shae.V2 term."). Statistically significant differences among distributions (independent 2-group Mann-Whitney U test) are indicated for $p \leq 0.001$ (***).

Click here to access/download
Supplementary Material
Supplementary_Table_S1.xlsx

Rejoinder R1

Click here to access/download;Personal Cover;Rejoinder_Sh_V2_GigaScience_R1_submit.docx

Dr Hongling Zhou
Junior Editor
GigaScience

25 June 2019

Dear Dr Zhou,

RE: GIGA-D-19-00167.R1

Herewith please find the R1 manuscript entitled "High-quality *Schistosoma haematobium* genome achieved by single-molecule and long-range sequencing" by Stroehlein et al., submitted for publication in *GigaScience*.

We thank you and the two reviewers for their positive and constructive reports on our manuscript. We are pleased that both reviewers are of the opinion that the improved genome assembly represents a very important resource for the research community and that it will serve as a useful reference for future investigations.

The present rejoinder addresses the reviewers' suggestions and recommendations (responses in bold-type) in a point-by-point manner:

Reviewer #1:

1.1. In the Background (line 101-109), the group mentioned the complex nature of its genome (repeats and segmental duplication) that was limiting a proper assembly structure using short reads. Such as *S. mansoni*, the annotation is also challenging since the gene structure of these worms are different from model organisms, for example, *S. mansoni* has lots of mini-exons (some with 3-6 nucleotide) with large introns. Without evidence, most of these are missed and not well detected by *ab initio* approaches. I would recommend adding something talking about this complexity, since the group did also a re-annotation of its genome.

RESPONSE: We have included a sentence on the challenges of gene prediction in non-model organisms with unique gene structures and have provided relevant references pertaining to schistosomes.

1.2. Line 184: add scalable nucleotide alignment Program (SNAP-align), Since you also use another tool called SNAP (Semi-HMM-based Nucleic Acid Parser - line 247) for annotation.

RESPONSE: We thank the reviewer for raising this point. We have amended the text to clarify that these are two distinct programs.

1.3. Line 199-202: the group was correct to fill gaps using PBJELLY using PacBio reads and scaffolding using SSPACE-LongRead. But there are two steps needed to make sure that the best possible assembly was produced. Long-read technologies unfortunately still have problems in the base call quality and a 19x fold would not be enough to correct all possible errors, so using the already

generated Illumina sequence from Shae.V1 the group could fill gaps or extend contigs using Image (PMID: 22678431) and then polishing erroneous base calls using ICORN2 (PMID: 22678431) or Pilon (PMID: 25409509).

RESPONSE: Reviewer #1 suggests two additional steps to improve assembly using Illumina reads: (i) error-correction of PacBio reads and (ii) gap-filling. We wish to emphasise that both of the suggestions have already been included in our analysis and are described in the *Methods* section. Therefore, we believe that no additional steps are needed to obtain the best possible assembly. As described in the *Methods* section, for "pre-assembly processing of sequencing data", we used published Illumina short-read libraries to error-correct the PacBio reads (referred to as "corrected PacBio reads" later on in the manuscript), employing the program LoRDEC (v.0.3). In addition, we have carried out steps to improve scaffolding and to close gaps using the same Illumina data sets employing Haplomerger 2 (v.3.2), as described in "Genome Assembly (2)". We have amended the text of these sections to further clarify the error-correction process.

1.4. Line 221-277: As I mentioned above, this genome has an intriguing annotation, *S. mansoni* for example is being revisited all the time and just now using IsoSeq from PacBio, they are finally finding new evidences for mini-exons and isoforms on its genome annotation. I didn't see any phrase specifying if this was done (using Artemis or Webapollo), where you can indeed check if you have enough support from different resources of this gene. I'm concerned of not doing this and just keeping the "longest ORF" since *Schistosoma* biology does not necessarily follow this assumption.

RESPONSE: We agree with Reviewer #1's opinion that schistosomes have an intriguing genome and are aware of the many fascinating characteristics yet to be explored. However, as stated in the *Discussion* section, the goal of the present study was to provide a *high-quality genomic resource* for *S. haematobium* to enable in-depth gene (re-)annotation, employing short- and long-read RNA-Seq data sets in the near future. In this context, a detailed re-annotation of the entire genome was beyond the scope of the present study. The gene annotation inferred here was used principally to evaluate the quality of the assembly, genome synteny and for comparative analyses. In our experience, using currently available short-read RNA-Seq data does not allow transcript isoforms to be accurately inferred, but reveals similar isoforms that differ only by UTR length, instead of accurately resolving alternative usage of internal exons. Detailed re-annotation using long-read RNA sequencing, such as PacBio IsoSeq and/or Oxford Nanopore Direct RNA sequencing, should allow us to resolve many of these features in the future, using the high-quality genomic resources presented here.

1.5. Line 265-277: When keeping the "longest ORF" you mean the whole transcript (all exons). If yes, maybe you need to change this to transcript, if not, I'm a bit worried if doing this is the best approach to get the right answer to your problem (see comment above talking about mini-exons).

RESPONSE: We have elected to use the longest open-reading frame as the representative coding region of a gene, because the objective here was to accurately capture the protein-coding regions and their boundaries for all genes, rather than resolving all possible combinations of exons (transcript isoforms). This aspect is still challenging and inaccurate due to the lack of long-read RNA-Seq data. Technologies producing such data have only recently become available and have improved enough in accuracy to facilitate such analyses in the near future. We thank the reviewer for their suggestion and agree that such technologies should be used in future re-annotation efforts of the enhanced genome of *S. haematobium*.

1.6. Line 299-309: Which substitution model was used for the ML tree reconstruction? Add this information and the analysis of ModelTest (which predicts the best substitution model should be used in your ML/BI analysis).

RESPONSE: We have used PartitionFinder (v.2.1.1) to establish the best substitution models and associated replacement matrices for each partition, as described in the *Methods* section. The

configuration files (raxml.txt and mrbayes.txt) for the ML and BI trees included in the GigaDB repository linked to the manuscript and contain all of the requested information.

1.7. Line 341-342: the 25 and 100 length sizes gaps are unknown gaps generated by PBJelly and HiRIse. PBJelly add 25 N bases when it can't be filled and HiRise follows the 100 bp gap window as requested by the community for unknown gap sizes. Usually you need to say this during the process of submission to NCBI, where all 100 bp N sites are considered unknown and other sizes are supported by physical mapping. This needs to be fixed. If you have any physical mapping supporting these gap sizes this needs to be better described in the manuscript (maybe with a supplementary figure).

RESPONSE: These gap sizes of 25 bp and 100 bp are introduced by the programs PBJelly and HiRise, respectively, as pointed out by the reviewer. We have added this information. Gap sizes of > 100 bp are estimated based on paired-end read mapping. Thus, we have set the "minimum number of N's in a row that represents a gap of estimated length" to 101 for the genome submission to NCBI. Although we cannot provide estimates for the unknown gap sizes introduced by PBJelly and HiRise, our coverage analysis (methods described in the last paragraph of the "Genome Assembly" section) showed that scaffolds containing gaps have a 'physical' coverage of ≥ 10 reads (i.e. spanned by ≥ 10 paired-end reads) (Figure 3), thus supporting correct scaffolding.

1.8. Line 125: add the name of the strain or isolate (e.g. MP2012). This helps other research groups in text mining analysis

RESPONSE: There is no name for the strain other than the description that we have provided. However, we have created individual BioSample IDs for the worms isolated in 2012 (used for sequencing for the first draft of the genome, designated MP2012) as well as those isolated and used for PacBio sequencing in the present manuscript (designated MP2018). We have amended the text and included the isolate and BioSample IDs to reflect the origin of those two samples.

1.9. Line 153: add version of the SMRT analysis software used for assembly (SMRTlink is the most recent)

RESPONSE: We have added the version information for the SMRT analysis software.

1.10. Line 123-309: Add versions to all software used, some like samtools (line 190) are missing.

RESPONSE: We have added all missing version information.

1.11. Line 288-292: Add a supplementary table with information about these parasites (with accession number) to help to know which version was used.

RESPONSE: Sequence data was obtained from WormBase ParaSite (WBPS13) or NCBI (where data had not been uploaded to WormBase). We have added this information and accession numbers for all parasite genome data sets used for the construction of phylogenetic trees.

1.12. Line 304: "1,000 bootstrap generations";

RESPONSE: This sentence describes the Bayesian inference analysis, which employs Markov Chain Monte Carlo sampling and not 'bootstrapping' employed by Maximum Likelihood-based tree-building methods (see PMID: 12598693). The support values for the BI analysis are not 'bootstrap support' values, but rather posterior probability (BPP) values. Thus, there is no need to alter the text in the manuscript.

1.13. Whole manuscript: fix inconsistencies: numbers, sometimes 1000 some times 1,000. BP, KB, MB and GB or nt. I recommend the first one.

RESPONSE: We have amended the manuscript accordingly. For numbers of ≥ 10,000, we have used a ‘thousands delimiter’ to improve readability of large numbers.

1.14. Suggestion for future work: Try to use Hi-C in a future re-assembly project, their sizes are much bigger than Chicago and will help to improve even more your results.

RESPONSE: We thank the reviewer for this suggestion. We will continue to adopt new state-of-the-art sequencing technologies to further improve the genome assembly and annotation in the future.

Reviewer #2:

2.1. Although discussed throughout the main body of the manuscript, further emphasis is required in the abstract to reflect that the sequencing carried out as part of this study was not used to assemble a wholly new version of the genome but instead was used to improve the version 1 assembly. The gene model mapping should also be included.

RESPONSE: The *Abstract* clearly summarises previous work done to obtain a first draft genome and mentions its limitations. This description is followed by “Here, we systematically enhanced the draft genome of *S. haematobium*…”. In our opinion, this sentence reflects the fact that the present study reports an improved genome, which is based on a published draft genome and new data sets created in this body of work. Therefore, we see no reason to alter the *Abstract*. Regarding “gene model mapping”, we have amended a sentence in the *Abstract* to indicate that the gene models were both transferred from the original gene set and predicted using improved gene annotation pipelines.

2.2. Line 182: can the authors provide further clarification regarding the unscaffolded contigs used in the Dovetail HiRise pipeline - were these the contigs used for the version 1 scaffold assembly or the contigs that did not scaffold in the original version?

RESPONSE: The un-scaffolded contigs were the contigs assembled for Shae.V1, before scaffolding. We have amended this sentence to clarify this aspect.

2.3. Lines 288-292: where are the genomes publicly available? If WormBase ParaSite, mention which version of the genomes were used for the phylogenetic analysis.

RESPONSE: Genome data were obtained from WormBase ParaSite WBPS13 and NCBI. We have clarified this aspect in the manuscript.

CONCLUDING REMARKS

We thank all reviewers for their time, effort and reports, and you for the time that you have spent handling and appraising the manuscript. We have revised the manuscript to address all of the reviewers’ comments (changes in the revised (R1) manuscript are highlighted in yellow). The suggested changes and recommendations have helped us in our revision. In our opinion, the revised manuscript now meets the standard for publication in *GigaScience*.

Yours sincerely,

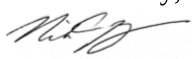

Neil D. Young PhD
NHMRC Career Development Fellow
Faculty of Veterinary and Agricultural Sciences, The University of Melbourne | Corner Flemington Road & Park Drive, Parkville, Victoria 3010, Australia | nyoung@unimelb.edu.au
