# Supplementary material for: High-quality Schistosoma haematobium genome achieved by single-molecule and long-range sequencing
Source: Gigascience. 2019 Sep 5;8(9):giz108. doi: 10.1093/gigascience/giz108 (PMC6736295; doi:10.1093/gigascience/giz108)
Supplement: giz108_Response_to_Reviewer_Comments_Original_Submission [file giz108_response_to_reviewer_comments_original_submission.pdf]

Author's Response To Reviewer Comments

Close

Dr Hongling Zhou
Junior Editor
GigaScience

25 June 2019

Dear Dr Zhou,

RE: GIGA-D-19-00167.R1

Herewith please find the R1 manuscript entitled "High-quality Schistosoma haematobium genome achieved by single-molecule and long-range sequencing" by Stroehlein et al., submitted for publication in GigaScience.

We thank you and the two reviewers for their positive and constructive reports on our manuscript. We are pleased that both reviewers are of the opinion that the improved genome assembly represents a very important resource for the research community and that it will serve as a useful reference for future investigations.

The present rejoinder addresses the reviewers' suggestions and recommendations (responses in bold-type) in a point-by-point manner:

Reviewer #1:

1.1. In the Background (line 101-109), the group mentioned the complex nature of its genome (repeats and segmental duplication) that was limiting a proper assembly structure using short reads. Such as S. mansoni, the annotation is also challenging since the gene structure of these worms are different from model organisms, for example, S. mansoni has lots of mini-exons (some with 3-6 nucleotide) with large introns. Without evidence, most of these are missed and not well detected by ab initio approaches. I would recommend adding something talking about this complexity, since the group did also a re-annotation of its genome.

RESPONSE: We have included a sentence on the challenges of gene prediction in non-model organisms with unique gene structures and have provided relevant references pertaining to schistosomes.

1.2. Line 184: add scalable nucleotide alignment Program (SNAP-align), Since you also use another tool called SNAP (Semi-HMM-based Nucleic Acid Parser - line 247) for annotation.

RESPONSE: We thank the reviewer for raising this point. We have amended the text to clarify that these are two distinct programs.

1.3. Line 199-202: the group was correct to fill gaps using PBJELLY using PacBio reads and scaffolding using SSPACE-LongRead. But there are two steps needed to make sure that the best possible assembly was produced. Long-read technologies unfortunately still have problems in the base call quality and a 19x fold would not be enough to correct all possible errors, so using the already generated Illumina sequence from Shae.V1 the group could fill gaps or extend contigs using Image (PMID: 22678431) and then polishing erroneous base calls using ICORN2 (PMID: 22678431) or Pilon (PMID: 25409509).

RESPONSE: Reviewer #1 suggests two additional steps to improve assembly using Illumina reads: (i) error-correction of PacBio reads and (ii) gap-filling. We wish to emphasise that both of the suggestions have already been included in our analysis and are described in the Methods section. Therefore, we believe that no additional steps are needed to obtain the best possible assembly. As described in the Methods section, for "pre-assembly processing of sequencing data", we used published Illumina short-

read libraries to error-correct the PacBio reads (referred to as “corrected PacBio reads” later on in the manuscript), employing the program LoRDEC (v.0.3). In addition, we have carried out steps to improve scaffolding and to close gaps using the same Illumina data sets employing Haplomerger 2 (v.3.2), as described in “Genome Assembly (2)”. We have amended the text of these sections to further clarify the error-correction process.

1.4. Line 221-277: As I mentioned above, this genome has an intriguing annotation, S. mansoni for example is being revisited all the time and just now using IsoSeq from PacBio, they are finally finding new evidences for mini-exons and isoforms on its genome annotation. I didn't see any phrase specifying if this was done (using Artemis or Webapollo), where you can indeed check if you have enough support from different resources of this gene. I'm concerned of not doing this and just keeping the "longest ORF" since Schistosoma biology does not necessarily follow this assumption.

RESPONSE: We agree with Reviewer #1’s opinion that schistosomes have an intriguing genome and are aware of the many fascinating characteristics yet to be explored. However, as stated in the Discussion section, the goal of the present study was to provide a high-quality genomic resource for S. haematobium to enable in-depth gene (re-)annotation, employing short- and long-read RNA-Seq data sets in the near future. In this context, a detailed re-annotation of the entire genome was beyond the scope of the present study. The gene annotation inferred here was used principally to evaluate the quality of the assembly, genome synteny and for comparative analyses. In our experience, using currently available short-read RNA-Seq data does not allow transcript isoforms to be accurately inferred, but reveals similar isoforms that differ only by UTR length, instead of accurately resolving alternative usage of internal exons. Detailed re-annotation using long-read RNA sequencing, such as PacBio IsoSeq and/or Oxford Nanopore Direct RNA sequencing, should allow us to resolve many of these features in the future, using the high-quality genomic resources presented here.

1.5. Line 265-277: When keeping the "longest ORF" you mean the whole transcript (all exons). If yes, maybe you need to change this to transcript, if not, I'm a bit worried if doing this is the best approach to get the right answer to your problem (see comment above talking about mini-exons).

RESPONSE: We have elected to use the longest open-reading frame as the representative coding region of a gene, because the objective here was to accurately capture the protein-coding regions and their boundaries for all genes, rather than resolving all possible combinations of exons (transcript isoforms). This aspect is still challenging and inaccurate due to the lack of long-read RNA-Seq data. Technologies producing such data have only recently become available and have improved enough in accuracy to facilitate such analyses in the near future. We thank the reviewer for their suggestion and agree that such technologies should be used in future re-annotation efforts of the enhanced genome of S. haematobium.

1.6. Line 299-309: Which substitution model was used for the ML tree reconstruction? Add this information and the analysis of ModelTest (which predicts the best substitution model should be used in your ML/BI analysis).

RESPONSE: We have used PartitionFinder (v.2.1.1) to establish the best substitution models and associated replacement matrices for each partition, as described in the Methods section. The configuration files (raxml.txt and mrbayes.txt) for the ML and BI trees included in the GigaDB repository linked to the manuscript and contain all of the requested information.

1.7. Line 341-342: the 25 and 100 length sizes gaps are unknown gaps generated by PBJelly and HiRIse. PBJelly add 25 N bases when it can’t be filled and HiRise follows the 100 bp gap window as requested by the community for unknown gap sizes. Usually you need to say this during the process of submission to NCBI, where all 100 bp N sites are considered unknown and other sizes are supported by physical mapping. This needs to be fixed. If you have any physical mapping supporting these gap sizes this needs to be better described in the manuscript (maybe with a supplementary figure).

RESPONSE: These gap sizes of 25 bp and 100 bp are introduced by the programs PBJelly and HiRise, respectively, as pointed out by the reviewer. We have added this information. Gap sizes of > 100 bp are estimated based on paired-end read mapping. Thus, we have set the “minimum number of N's in a row that represents a gap of estimated length” to 101 for the genome submission to NCBI. Although we cannot provide estimates for the unknown gap sizes introduced by PBJelly and HiRise, our coverage analysis (methods described in the last paragraph of the “Genome Assembly” section) showed that scaffolds containing gaps have a ‘physical’ coverage of ≥ 10 reads (i.e. spanned by ≥ 10 paired-end

reads) (Figure 3), thus supporting correct scaffolding.

1.8. Line 125: add the name of the strain or isolate (e.g. MP2012). This helps other research groups in text mining analysis

RESPONSE: There is no name for the strain other than the description that we have provided. However, we have created individual BioSample IDs for the worms isolated in 2012 (used for sequencing for the first draft of the genome, designated MP2012) as well as those isolated and used for PacBio sequencing in the present manuscript (designated MP2018). We have amended the text and included the isolate and BioSample IDs to reflect the origin of those two samples.

1.9. Line 153: add version of the SMRT analysis software used for assembly (SMRTlink is the most recent)

RESPONSE: We have added the version information for the SMRT analysis software.

1.10. Line 123-309: Add versions to all software used, some like samtools (line 190) are missing.

RESPONSE: We have added all missing version information.

1.11. Line 288-292: Add a supplementary table with information about these parasites (with accession number) to help to know which version was used.

RESPONSE: Sequence data was obtained from WormBase ParaSite (WBPS13) or NCBI (where data had not been uploaded to WormBase). We have added this information and accession numbers for all parasite genome data sets used for the construction of phylogenetic trees.

1.12. Line 304: "1,000 bootstrap generations";

RESPONSE: This sentence describes the Bayesian inference analysis, which employs Markov Chain Monte Carlo sampling and not ‘bootstrapping’ employed by Maximum Likelihood-based tree-building methods (see PMID: 12598693). The support values for the BI analysis are not ‘bootstrap support’ values, but rather posterior probability (BPP) values. Thus, there is no need to alter the text in the manuscript.

1.13. Whole manuscript: fix inconsistencies: numbers, sometimes 1000 some times 1,000. BP, KB, MB and GB or nt. I recommend the first one.

RESPONSE: We have amended the manuscript accordingly. For numbers of ≥ 10,000, we have used a ‘thousands delimiter’ to improve readability of large numbers.

1.14. Suggestion for future work: Try to use Hi-C in a future re-assembly project, their sizes are much bigger than Chicago and will help to improve even more your results.

RESPONSE: We thank the reviewer for this suggestion. We will continue to adopt new state-of-the-art sequencing technologies to further improve the genome assembly and annotation in the future.

Reviewer #2:

2.1. Although discussed throughout the main body of the manuscript, further emphasis is required in the abstract to reflect that the sequencing carried out as part of this study was not used to assemble a wholly new version of the genome but instead was used to improve the version 1 assembly. The gene model mapping should also be included.

RESPONSE: The Abstract clearly summarises previous work done to obtain a first draft genome and mentions its limitations. This description is followed by “Here, we systematically enhanced the draft genome of S. haematobium…”. In our opinion, this sentence reflects the fact that the present study reports an improved genome, which is based on a published draft genome and new data sets created in this body of work. Therefore, we see no reason to alter the Abstract. Regarding “gene model mapping”, we have amended a sentence in the Abstract to indicate that the gene models were both transferred from the original gene set and predicted using improved gene annotation pipelines.

2.2. Line 182: can the authors provide further clarification regarding the unscaffolded contigs used in the Dovetail HiRise pipeline - were these the contigs used for the version 1 scaffold assembly or the contigs that did not scaffold in the original version?

RESPONSE: The un-scaffolded contigs were the contigs assembled for Shae.V1, before scaffolding. We have amended this sentence to clarify this aspect.

2.3. Lines 288-292: where are the genomes publicly available? If WormBase ParaSite, mention which version of the genomes were used for the phylogenetic analysis.

RESPONSE: Genome data were obtained from WormBase ParaSite WBPS13 and NCBI. We have clarified this aspect in the manuscript.

____

CONCLUDING REMARKS
We thank all reviewers for their time, effort and reports, and you for the time that you have spent handling and appraising the manuscript. We have revised the manuscript to address all of the reviewers' comments (changes in the revised (R1) manuscript are highlighted in yellow). The suggested changes and recommendations have helped us in our revision. In our opinion, the revised manuscript now meets the standard for publication in GigaScience.

Yours sincerely,

Neil D. Young PhD
NHMRC Career Development Fellow
Faculty of Veterinary and Agricultural Sciences, The University of Melbourne | Corner Flemington Road & Park Drive, Parkville, Victoria 3010, Australia | nyoung@unimelb.edu.au

Close
