# Supplementary material for: High-quality Schistosoma haematobium genome achieved by single-molecule and long-range sequencing
Source: Gigascience. 2019 Sep 5;8(9):giz108. doi: 10.1093/gigascience/giz108 (PMC6736295; doi:10.1093/gigascience/giz108)
Supplement: giz108_Reviewer_1_Report_Original_Submission — Rodrigo Baptista, Ph.D. -- 5/24/2019 Reviewed [file giz108_reviewer_1_report_original_submission.pdf]

Reviewer Report

Title: High-quality Schistosoma haematobium genome achieved by single-molecule and long-range sequencing

Version: Original Submission **Date:** 5/24/2019

Reviewer name: Rodrigo Baptista, Ph.D.

Reviewer Comments to Author:

The research group did a excellent job re-assembling and re-annotating the S. haematobium draft genome, which is a very complex genome and considered a challenge for most research groups all over the world. The group goal to provide a high-quality genomic resource for S. haematobium was achieved and this was made using most of the genome assembly good practices recommended by the community. These results will indeed serve as a reference for future work with not just this worm parasite, but also for related species, enabling biological investigations and possible disease interventions.
Besides agreeing with all analysis performed by the group there are a few recommendations and suggestions to improve the manuscript:
Major:
- In the Background (line 101-109), the group mentioned the complex nature of its genome (repeats and segmental duplication) that was limiting a proper assembly structure using short reads. Such as S. mansoni, the annotation is also challenging since the gene structure of these worms are different from model organisms, for example, S. mansoni has lots of mini-exons (some with 3-6 nucleotide) with large introns. Without evidence, most of these are missed and not well detected by ab-initio approaches. I would recommend to add something talking about this complexity, since the group did also a re-annotation of its genome.
- line 184: add scalable nucleotide alignment Program (SNAP-align), Since you also use another tool called SNAP (Semi-HMM-based Nucleic Acid Parser - line 247) for annotation.
- line 199-202: the group was correct to fill gaps using PBJELLY using Pacbio reads and scaffolding using SSPACE-LongRead. But there are two steps needed to make sure that the best possible assembly was produced. Long-read technologies unfortunately still have problems in the base call quality and a 19x fold would not be enough to correct all possible errors, so using the already generated Illumina sequence from ShaeV1 the group could fill gaps or extend contigs using Image (PMID: 22678431) and then polishing erroneous base calls using ICORN2 (PMID: 22678431) or Pilon (PMID: 25409509).
- line 221-277: As I mentioned above this genome has a intriguing annotation, S. mansoni for example is being revisited all the time and just now using IsoSeq from Pacbio they are finally finding new evidences for mini-exons and isoforms on its genome annotation. I didn't saw any phrase specifying if this was done (using Artemis or Webapollo), where you can indeed check if you have enough support from different resources of this gene. I'm concern of not doing this and just keeping the "longest ORF" since Schisto biology does not follow necessary this assumption.
- line 265-277: When keeping the "longest ORF" you mean the whole transcript (all exons). If yes,

maybe you need to change this to transcript, if not, I'm a bit worried if doing this is the best approach to get the right answer to your problem (see comment above talking about mini-exons)
- line 299-309: Which substitution model was used for the ML tree reconstruction? Add this information and the analysis of ModelTest (which predicts the best substitution model should be used in your ML/BI analysis).
- line 341-342: the 25 and 100 length sizes gaps are unknown gaps generated by PBJelly and HiRIse. PBJelly add 25 N bases when it cant be filled and HiRise follows the 100 bp gap window as requested by the community for unknown gap sizes. Usually you need to say this during the process of submission to NCBI, where all 100 bp N sites are considered unknown and other sizes are supported by physical mapping. This needs to be fixed. If you have any physical mapping supporting these gap sizes this needs to be better described in the manuscript (maybe with a supplementary figure).
Minor:
- line 125: add the name of the strain or isolate (e.g. MP2012). This helps other research groups in text mining analysis
- line 153: add version of the SMRT analysis software used for assembly (SMRTlink is the most recent)
- line 123-309: Add versions to all software used, some like samtools (line 190) are missing.
- line 288-292: Add a supplementary table with information about these parasites (with accession number) to help to know which version was used.
- line 304: "1,000 bootstrap generations";
- whole manuscript: fix inconsistencies:
    - Numbers, sometimes 1000 some times 1,000
    - BP, KB, MB and GB or nt. I recommend the first one.
Suggestion for future work:
- Try to use Hi-C in a future re-assembly project, their sizes are much bigger than Chicago and will help to improve even more your results.

Level of Interest

Please indicate how interesting you found the manuscript: Choose an item.

Quality of Written English

Please indicate the quality of language in the manuscript: Choose an item.

Declaration of Competing Interests

Please complete a declaration of competing interests, considering the following questions:

- Have you in the past five years received reimbursements, fees, funding, or salary from an organisation that may in any way gain or lose financially from the publication of this manuscript, either now or in the future?

- Do you hold any stocks or shares in an organisation that may in any way gain or lose financially from the publication of this manuscript, either now or in the future?
- Do you hold or are you currently applying for any patents relating to the content of the manuscript?
- Have you received reimbursements, fees, funding, or salary from an organization that holds or has applied for patents relating to the content of the manuscript?
- Do you have any other financial competing interests?
- Do you have any non-financial competing interests in relation to this paper?

If you can answer no to all of the above, write 'I declare that I have no competing interests' below. If your reply is yes to any, please give details below.

I declare that I have no competing interests

I agree to the open peer review policy of the journal. I understand that my name will be included on my report to the authors and, if the manuscript is accepted for publication, my named report including any attachments I upload will be posted on the website along with the authors' responses. I agree for my report to be made available under an Open Access Creative Commons CC-BY license (http://creativecommons.org/licenses/by/4.0/). I understand that any comments which I do not wish to be included in my named report can be included as confidential comments to the editors, which will not be published.

Choose an item.

To further support our reviewers, we have joined with Publons, where you can gain additional credit to further highlight your hard work (see: https://publons.com/journal/530/gigascience). On publication of this paper, your review will be automatically added to Publons, you can then choose whether or not to claim your Publons credit. I understand this statement.

Yes Choose an item.
