# Supplementary material for: High-quality Schistosoma haematobium genome achieved by single-molecule and long-range sequencing
Source: Gigascience. 2019 Sep 5;8(9):giz108. doi: 10.1093/gigascience/giz108 (PMC6736295; doi:10.1093/gigascience/giz108)
Supplement: giz108_Reviewer_2_Report_Original_Submission — Krystyna Cwiklinski -- 6/5/2019 Reviewed [file giz108_reviewer_2_report_original_submission.pdf]

Reviewer Report

Title: High-quality Schistosoma haematobium genome achieved by single-molecule and long-range sequencing

Version: Original Submission **Date:** 6/5/2019

Reviewer name: Krystyna Cwiklinski

Reviewer Comments to Author:

The manuscript by Stroehlein and colleagues reports the improved genome assembly for the blood fluke Schistosoma haematobium. This study complements the sequence data currently available for Schistosoma parasites. In particular, this study is enhanced by the methodology used by the authors to use the single molecule and long-range sequence data to improve upon the current S. haematobium assembly and to consolidate the gene model information. This allows research continuity providing an important future resource.
The manuscript is well written and is suitable for publication in GigaScience as a data note following minor clarification/revision.
1. Although discussed throughout the main body of the manuscript, further emphasis is required in the abstract to reflect that the sequencing carried out as part of this study was not used to assemble a wholly new version of the genome but instead was used to improve the version 1 assembly. The gene model mapping should also be included.
2. Line 182 - can the authors provide further clarification regarding the unscaffolded contigs used in the Dovetail HiRise pipeline - were these the contigs used for the version 1 scaffold assembly or the contigs that did not scaffold in the original version?
3. Lines 288-292 - where are the genomes publicly available? If WormBase ParaSite, mention which version of the genomes were used for the phylogenetic analysis.

Level of Interest

Please indicate how interesting you found the manuscript: Choose an item.

Quality of Written English

Please indicate the quality of language in the manuscript: Choose an item.

Declaration of Competing Interests

Please complete a declaration of competing interests, considering the following questions:

- Have you in the past five years received reimbursements, fees, funding, or salary from an organisation that may in any way gain or lose financially from the publication of this manuscript, either now or in the future?
- Do you hold any stocks or shares in an organisation that may in any way gain or lose financially from the publication of this manuscript, either now or in the future?
- Do you hold or are you currently applying for any patents relating to the content of the manuscript?
- Have you received reimbursements, fees, funding, or salary from an organization that holds or has applied for patents relating to the content of the manuscript?
- Do you have any other financial competing interests?
- Do you have any non-financial competing interests in relation to this paper?

If you can answer no to all of the above, write 'I declare that I have no competing interests' below. If your reply is yes to any, please give details below.

I declare that I have no competing interests.

I agree to the open peer review policy of the journal. I understand that my name will be included on my report to the authors and, if the manuscript is accepted for publication, my named report including any attachments I upload will be posted on the website along with the authors' responses. I agree for my report to be made available under an Open Access Creative Commons CC-BY license (http://creativecommons.org/licenses/by/4.0/). I understand that any comments which I do not wish to be included in my named report can be included as confidential comments to the editors, which will not be published.

Choose an item.

To further support our reviewers, we have joined with Publons, where you can gain additional credit to further highlight your hard work (see: https://publons.com/journal/530/gigascience). On publication of this paper, your review will be automatically added to Publons, you can then choose whether or not to claim your Publons credit. I understand this statement.

Yes Choose an item.
